# “Exact” and Approximate Methods for Bayesian Inference: Stochastic Volatility Case Study

**DOI:** 10.3390/e23040466

**Published:** 2021-04-15

**Authors:** Yuliya Shapovalova

**Affiliations:** Institute for Computing and Information Sciences, Radboud University Nijmegen, Toernooiveld 212, 6525 EC Nijmegen, The Netherlands; yuliya.shapovalova@ru.nl

**Keywords:** Bayesian inference, Markov Chain Monte Carlo, Sequential Monte Carlo, Riemann Manifold Hamiltonian Monte Carlo, integrated nested laplace approximation, fixed-form variational Bayes, stochastic volatility

## Abstract

We conduct a case study in which we empirically illustrate the performance of different classes of Bayesian inference methods to estimate stochastic volatility models. In particular, we consider how different particle filtering methods affect the variance of the estimated likelihood. We review and compare particle Markov Chain Monte Carlo (MCMC), RMHMC, fixed-form variational Bayes, and integrated nested Laplace approximation to estimate the posterior distribution of the parameters. Additionally, we conduct the review from the point of view of whether these methods are (1) easily adaptable to different model specifications; (2) adaptable to higher dimensions of the model in a straightforward way; (3) feasible in the multivariate case. We show that when using the stochastic volatility model for methods comparison, various data-generating processes have to be considered to make a fair assessment of the methods. Finally, we present a challenging specification of the multivariate stochastic volatility model, which is rarely used to illustrate the methods but constitutes an important practical application.

## 1. Introduction

The field of Bayesian statistics and machine learning has advanced in recent years quite rapidly. The methods that have been developed do not often find fast assimilation across different fields. In this review, we aim to provide the reader with methodologies that try to solve the estimation problem in models with latent variables and intractable likelihoods. We are in particular interested in the methods that can be used to estimate nonlinear state-space models and in particular stochastic (latent) volatility models. There are multiple studies that conducted review and comparison of the methods of estimation of the stochastic volatility models [1,2,3]. We briefly mention some of the methods that have been reviewed; however, most of the methods considered in this paper have not entered those reviews. In this paper, we focus in particular on comparing methods that target posterior distribution exactly and the methods that try to approximate it. We also conduct the review from the point of view of estimating multivariate models with these methods and discuss what the bottleneck is in each of them when extending to higher-dimensional stochastic volatility (SV) models. We consider different data-generating processes for simulating data in the empirical studies and conclude that the choice of the data-generating process can heavily affect performance of a method. Thus, illustrating the performance of a method on just one data generating process or one real-world data set is not sufficient.

In financial econometrics literature, GARCH-type models prevail since they are much simpler to estimate. Stochastic (latent) volatility models, however, can be more natural frameworks for modeling asset returns. They can provide flexible and intuitive tools for applications in financial econometrics as well as some other disciplines. In particular, multivariate stochastic volatility models offer an attractive framework for detection and measuring *volatility spillover effects*. Volatility spillovers in this framework can be defined through Granger-causal links in the latent (unobservable) volatility process, which is modeled with a Vector Autoregressive model (VAR(p)). Insights about the causal structure can help to identify the relationship (Granger-causality or/and contemporaneous correlation) between the financial markets. Such information can be insightful and helpful in the decision-making process of portfolio managers and policymakers. These models are, however, rarely considered in practice. Multiple Bayesian inference methods have been proposed for the estimation of this class of models in recent years. In this paper, we identify the bottlenecks in different classes of methods for the estimation of these models in the multivariate case.

One of the stepping stones of estimation of the nonlinear state-space models in general (and stochastic volatility models in particular) lies in the intractability of the likelihood, which is the result of the presence of an unobservable process in the model and nonlinear dependence between this process and the observed data. The likelihood can be estimated with particle filter methods, also known as Sequential Monte Carlo. This is a computationally intensive procedure; however, depending on the problem and the data, it can provide excellent results. The second stepping stone of the estimation is the intractable posterior distribution. A standard starting point for sampling from the posterior distribution is the Metropolis-Hastings algorithm, which is a general method and can be applied straightforwardly to different models. It works well when the number of parameters in the model is small. However, the convergence of the algorithm can be slow in larger models, due to inefficiency of the sampling with random walk proposals. Particle Metropolis-Hastings [4] combines Sequential Monte Carlo for the likelihood estimation with Metropolis-Hastings for the sampling from the posterior, which results in a state-of-the-art method in terms of the estimation quality since it targets the exact posterior. The downside of this method is that it is computationally extremely demanding. Note that, while we consider particle Metropolis-Hastings in this paper, the class of methods from [4] is more general.

Two main downsides of particle Metropolis-Hastings are random walk behavior of the proposals and computational burden. One of the possible solutions to the first problem are the algorithms that use gradient information for the construction of the proposal distribution and thus explore the parameter space more efficiently. An additional step in improving these algorithms is defining them on a Riemann manifold instead of Euclidean space as proposed in [5]. The resulting algorithm, which we consider for the comparison in this paper, is Riemann Manifold Hamiltonian Monte Carlo. For extensive comparison of the methods that exploit gradient information and Langevin dynamics—such as Metropolis-adjusted Langevin algorithm, Hamiltonian Monte Carlo, Riemann manifold Metropolis adjusted Langevin algorithm, andRiemann Manifold Hamiltonian Monte Carlo—we refer to [5].

Thus far, we have discussed the methods that target the posterior distribution exactly and have a high computational burden, which makes empirical investigation of their performance in high-dimensional cases infeasible. In the last decade, a large number of methods have been published on approximate posterior inference thaat allow much faster computations, but lose in terms of precision of the estimation. In this paper, we consider two such methods that deal with different types of approximation. Fixed-form variational Bayes, proposed in [6], assumes hierarchical factorization of the prior and posterior distributions, and the factorized distributions are approximated by an analytically tractable distribution from a certain family of distributions q(·). Then, instead of solving integration problem, one solves the optimization problem of minimizing the Kullback–Leibler divergence between q(·) and p(·), where p(·) is the target distribution. The second approximate method that we consider is the integrated nested Laplace approximation (INLA) [7]. The method relies on the nested version of the classical Laplace approximation. It became very popular in recent years and made computations in many models feasible.

In this review paper, we focus our attention on the following methodologies and provide a comparison for some of the methods via a simulation study. We consider how the variance of the estimated likelihood is affected by choosing different particle-filtering algorithms. Unlike previous studies, we consider the variance of the estimated likelihood over the whole parameter space and notice that it is affected by some parameters of the model more than by the others. We compare particle Metropolis-Hastings with Riemann Manifold Hamiltonian Monte Carlo as two state-of-the-art sampling methods for this type of problem. We asses how well the INLA method performs in the task of the estimation of the parameters of stochastic volatility model and finally, compare fixed-form variational Bayes methods with sampling by RMHMC. All the between-methods comparisons are performed on multiple simulated data sets with different underlying parameters. We illustrate that, for fair comparison and performance assessment, illustration only on data sets is not sufficient.

The paper is organized as follows. In Section 2, we introduce the model and its different specifications. While in simulation studies we use univariate model, we do introduce multivariate stochastic volatility models with Granger-causal feedback as the model of interest for high-dimensional inference. In Section 3, we review the methods that can be used for the estimation of this class of models. We introduce major ideas behind these methods, and for the details of the derivations we refer to the original papers. In Section 4, we perform empirical case study on different simulated data sets and compare the methods on two real-world time series. We in particular focus on the precision loss of parameter estimation when using approximate methods and how adaptable the methods are to perform multivariate estimation and estimation of various model specifications.

## 2. Model

### 2.1. Univariate Stochastic Volatility Model

In this section, we introduce the model of interest that we will use in the simulation studies. Stochastic volatility (SV) models are concerned with modeling asset prices or asset returns depending on how the model is formulated. Let $P_{t}$ be the price of the asset at time *t* or the exchange rate at time *t* (we consider two applications to real data in Section 3.5: one to exchange rate and one to log-returns). Then the log-return $y_{t}$ is

(1) $$y_{t}=\log\left(1+R_{t}\right)=\log\frac{P_{t}}{P_{t-1}}.$$

Stochastic volatility models are built in such a way that they can mimic *stylized facts* about financial markets and log-returns $y_{t}$. Stylized facts are empirically observed statistical properties of asset prices and asset returns. Typical examples of stylized facts are

- *Volatility clustering and persistence*: the big changes in asset returns tend to be followed by big changes, and small changes in asset returns tend to be followed by small changes; in other words, there are periods of large fluctuations and small fluctuations [8].
- *Leverage effect*: the changes in stock prices may be negatively related to the changes in volatility [9].
- *Co-movements:* different stocks tend to exhibit co-movements, which means that if the volatility of one stock changes in a specific direction, volatilities of the other stocks tend to change in the same direction [9].

One of the earlier works that received much attention in the financial literature and proposed a mathematical model that tried to explain the dynamics of financial markets is [10]. Numerous continuous-time stochastic volatility models have been proposed since then, and among the first ones, multiple variants should be mentioned [11,12,13,14]. The model we will be considering in this chapter can be viewed as a discrete version of the model in [13] derived by using Euler–Maruyama approximation. The stochastic volatility model in continuous time can be written as

(2) $$\begin{array}{cc} \; & ds\left(t\right)=\sigma \left(t\right)dB_{1}\left(t\right), \end{array}$$

(3) $$\begin{array}{cc} \; & \ln\sigma^{2}\left(t\right)=\mu +\beta \ln\sigma^{2}\left(t\right)dt+\sigma_{\eta }dB_{2}\left(t\right), \end{array}$$

where s(t) is log of asset price, $\sigma^{2}\left(t\right)$ is the volatility, $B_{1}\left(t\right)$ and $B_{2}\left(t\right)$ are Brownian motions that satisfy $corr(B_{1}\left(t\right),B_{2}\left(t\right))=\rho$. If ρ<0, there is leverage effect present. Thus, log of asset price follows diffusion and its volatility parameter also follows diffusion [15]. As we often get the data in discrete time, usually the discrete time approximation of the model is used in practice. The discrete model then follows by using Euler–Maruyama approximation

(4) $$\begin{array}{cc} \; & y_{t}=\sigma_{t}\epsilon_{t}, \end{array}$$

(5) $$\begin{array}{cc} \; & \ln\sigma_{t+1}^{2}=\mu +\phi \ln\sigma_{t}^{2}+\sigma_{\eta }\eta_{t+1}, \end{array}$$

where $y_{t}$ is logarithmic return, $\epsilon_{t}=B_{1}\left(t+1\right)-B_{1}\left(t\right)$, $\eta_{t+1}=B_{2}\left(t+1\right)-B_{2}\left(t\right)$, ϕ=1+β. Further, $\epsilon_{t}∼N\left(0,1\right)$ and $\eta_{t}∼N\left(0,1\right)$, $corr(\epsilon_{t},\eta_{t+1})=\rho .$

**Figure 1:**
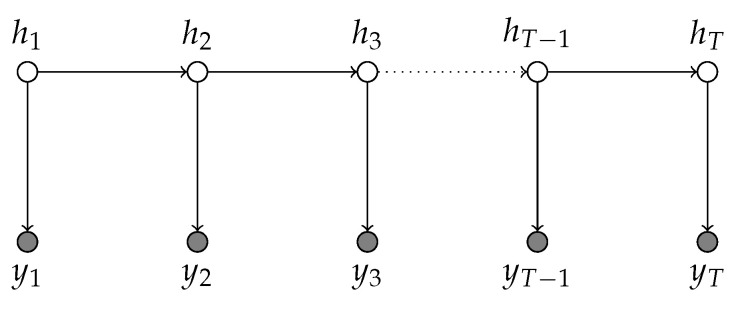
Graphical representation of stochastic volatility model. Observations $y_{t}$ represented by shaded edges depend at each time point on the state of the latent volatility process $h_{t}$.

We get state-space representation of the model that is commonly used by defining $h_{t}=\ln\sigma_{t}^{2}$ and $\sigma_{t}^{2}=\exp\left(h_{2}\right)$

(6) $$\begin{array}{cc} \; & y_{t}=\exp\left(h_{t}/2\right)\epsilon_{t}, \end{array}$$

(7) $$\begin{array}{cc} \; & h_{t+1}=\mu +\phi h_{t}+\eta_{t+1}, \end{array}$$

where $y_{t}$ are log-returns that are observed and volatility $h_{t}$ is latent and drives the dynamics of $y_{t}$. Figure 1 illustrates this structure of the model. Note that the latent volatility process has an autoregressive form. However, unlike in the standard autoregressive model, the latent volatility is not observed and thus has to be estimated together with the model parameters μ, ϕ, and $\sigma_{\eta }$, which are the scale, the volatility persistence and the noise variance of the latent volatility process, respectively. The persistence parameter ϕ reflects one of the stylized facts of financial returns, namely volatility persistence. The intuition is as follows: if ϕ>0 and $\exp(h_{t-1}/2)$ is large, then $\exp(h_{t}/2)$ will tend to be large too. Hence, the model can account for volatility clustering. In this paper, we consider stationary volatility cases with |ϕ|<1. Finally, one can also incorporate leverage effects by defining negative correlation between noise terms $\epsilon_{t}$ and $\eta_{t+1}$. Intuitive interpretation of the leverage effect goes as follows: bad news tends to decrease the price, which means that financial leverage increases, the firm becomes riskier, and thus expected volatility also increases. The leverage effects in this model have been studied in [16]. The stochastic volatility model can be parametrized in multiple ways; often, the following alternatives are considered [2]. Other ways to parametrize this model are presented in Equarions (8) and (9). The left-hand side version of the model corresponds to that of [17]. The right-hand side version is a different way to define the scaling parameter; in this case, it is β. For identifiability reasons, only β or μ as in Equation (7) should be included in the model.

(8) $$\begin{array}{cc} y_{t}=\sqrt{h_{t}}\epsilon_{t} & y_{t}=\beta \exp\left(h_{t}/2\right)\epsilon_{t} \end{array}$$

(9) $$\begin{array}{cc} \log h_{t}=\mu +\phi \log h_{t-1}+\eta_{t} & h_{t}=\phi h_{t-1}+\eta_{t}. \end{array}$$

Note that the authors of [17] define the leverage effect as correlation between $\epsilon_{t}$ and $\eta_{t}$, so the correlation between noise terms is contemporaneous while [16] model correlation between $\epsilon_{t}$ and $\eta_{t+1}$, which corresponds to correlation of the returns with one-step-ahead volatility. Reference Yu [18] shows that the approach of [16] is preferable. In particular, while in case of [16] the model is a martingale difference sequence, i.e., the past does not help to predict the future of the time series, in the case of [17], it is not. Hence, in the latter case, the efficient market hypothesis is violated.

In the remainder of this manuscript, we will work with either specification of the model defined in Equations (6) and (7) or in right-hand side of Equations (8) and (9). These models are equivalent, and we interchange the representation either for the convenience of using some of the methods or for comparison with other work. In the literature, both specifications are frequently used, and in some papers (for example, ref. [19]) the transition from one specification to another is conducted by observing that β=exp(μ/2).

Under the assumption that |ϕ|<1, the unconditional first and second moments of the latent process $h_{t}$ are

(10) $$E\left(h_{t}\right)=\frac{\mu }{1-\phi },\;Var\left(h_{t}\right)=\frac{\sigma_{\eta }^{2}}{1-\phi^{2}}.$$

The challenge of the estimation of the model lies in the intractability of the likelihood and posterior distribution. The likelihood factorizes as

(11) $$L\left(y|\theta \right)=\prod_{t=1}^{T}p\left(y_{t}|y_{1:t-1},\theta \right),$$

where the terms in the product can be computed recursively, and it becomes clear that the likelihood is a high-dimensional integral

(12) $$p\left(y_{t}|y_{1:t-1},\theta \right)=\int p\left(y_{t}|h_{t},\theta \right)p\left(h_{t}|y_{1:t-1},\theta \right)dh_{t}.$$

There is no analytical solution to the integral in Equation (12), and in this paper, we consider methods to estimate it using sequential Monte Carlo methods.

### 2.2. Multivariate Stochastic Volatility Model

In this section, we introduce the multivariate stochastic volatility model, which is rarely used in practice due to the challenges of estimation. One of the objectives of this paper is to assess whether modern methods in Bayesian inference are capable of the estimation of these models in high-dimensional case. Multivariate or high dimensional application of this class of models can give insightful information to practitioners. We deal with the same set-up as before; however, we now consider multiple time series of logarithmic returns that are interconnected through the latent volatility process

(13) $$y_{t}=\Omega_{t}\epsilon_{t},$$

where $\epsilon_{t}∼N\left(0,R\right)$ and R is a correlation matrix with entries $r_{ii}=1$, i=1,…,n on the diagonal. Furthermore, $\Omega_{t}$ is a diagonal matrix that contains time-varying volatilities that are driven by an independent stochastic process $h_{t}$,

$$\Omega_{t}=diag\left(\exp(h_{t}/2)\right).$$

The process $h_{t}$ of log-volatilities follows a VAR(*p*) process

(14) $$h_{t}=\mu +\sum_{k=1}^{p}\Phi_{k}\;\left(h_{t-i}-\mu \right)+\eta_{t},$$

where $\Phi_{k}={\left(\phi_{ij,k}\right)}_{i,j=1,\ldots ,n}$ are n×n coefficient matrices. Introducing the matrices $\Phi_{k}={\left(\phi_{ij,k}\right)}_{i,j=1,\ldots ,n}$ allows us to model connectivity in financial time series through the concept of Granger-causality in latent volatility process. We say that $h_{i}$ does not Granger-cause $h_{j}$ if all ${\left(\phi_{ij,k}\right)}_{k=1,\ldots ,p}=0$. The standard conditions on stationarity of a vector autoregressive model apply: the root of |I−λΦ|=0 should lie outside the unit circle, and the errors $\eta_{t}$ are independent and identically normally distributed with mean zero and variance-covariance matrix $\Sigma =diag(\sigma_{1}^{2},\ldots ,\sigma_{n}^{2})$. Equations (13) and  (14) are multivariate extensions of the model described in Equations (6) and (7). The representation from the right-hand side of Equations  (8) and (9) can be obtained by including a vector of parameters β into $\Omega_{t}$ and removing μ from Equation (14). As before, for identifiability, only one vector of the scale parameters—either μ or β—should be included in the model.

The above MSV model can also be viewed as a non-linear state-space model where (14) is the state equation of the latent process $h_{t}$ and (13) is the observation equation that depends non-linearly on the latent state. Note that, in this model, the time series are interconnected and the relationship between them can be interpreted through the concept of Granger-causality in latent volatility processes.

## 3. Methods

### 3.1. Bayesian Inference

In this paper, we review various methods that sample from or approximate the posterior distribution of the parameters of the model θ. The sampling or approximate methods are necessary since we are working in the framework when the posterior distribution and the likelihood are analytically intractable. The Bayes’ rule allows us to write posterior distribution in the form

(15) $$p\left(\theta |y\right)=\frac{\pi (\theta )g(y|\theta )}{m(y)},$$

where π(θ) is the prior distribution of the parameters of the model, g(y|θ) is the likelihood of the data given parameters of the model, and m(y) is the marginal density of *y*, which can be viewed as normalizing constant and which we will ignore in this paper. In the remainder of the paper we will work with the Bayes’ rule in proportionality terms:

(16) $$\begin{array}{c} p(\theta |y)\propto \pi (\theta )g(y|\theta ). \end{array}$$

Note that in the stochastic volatility model we have to estimate parameters of the model $\theta =(\mu ,\phi ,\sigma^{2})$ and the latent vector of volatilities h. Thus, we are interested in the following form of the Bayes’ rule

(17) p(θ,h|y)∝g(y|θ,h)f(h|θ)π(θ).

Multiple approaches can be used for the estimation of p(θ,h|y). One of the challenges is that neither posterior p(θ,h|y) nor the likelihood g(y|θ,h) is tractable. We start our review by considering sequential Monte Carlo methods, also known as particle filtering, for the estimation of the likelihood g(y|θ,h). We then discuss Metropolis-Hastings algorithm for sampling from the posterior and how these two algorithm can be combined into particle Metropolis-Hastings for sampling from the posterior distribution. We continue the review of the methods by considering RMHMC method in which the parameters and volatilities are sampled within the same framework. Finally, we review two approximate methods: integrated nested Laplace approdximation and fixed-form variational Bayes, two different ways of approximating posterior distribution.

### 3.2. Sequential Monte Carlo for the Estimation of the Likelihood

The Sequential Monte Carlo (SMC) method, also known in the literature as *particle filtering*, is considered a state-of-the-art method for estimation of the intractable likelihoods in nonlinear state-space models. The general idea behind this method lies in the estimation of the latent states by drawing multiple samples (particles) and then propagating them in time according to corresponding importance weights. By combining the weights over all time steps, one obtains a marginal likelihood estimate. Standard and well-known schemes are *Bootstrap particle filter* (BPF) [20], *Seqiential Importance Sampling* (SIS), and *Seqiential Importance Resampling* (SIR) [21]. Sequential Monte Carlo methods were elegantly combined with Markov Chain Monte Carlo in [4], and the method was named particle Markov Chain Monte Carlo (PMCMC). This method provides a powerful and coherent approach for Bayesian inference in a wide range of complex models. In the later subsections, we will discuss how sequential Monte Carlo methods are combined with Markov Chain Monte Carlo for fully Bayesian inference in stochastic volatility models. One of the concerns when using and implementing SMC for the likelihood estimation is the variance of the estimated likelihood. Standard SMC techniques such as SIS are prone to have high variance of the estimated likelihood once the dimensionality of the problem increases [22]. A number of studies have tried to address this problem. The common choice of proposal for sample of particles in standard schemes is $f(h_{t}|h_{t-1})$. Pitt and Shephard [23] propose an *auxiliary particle filter* as a solution that is using proposal for particles which takes into account the current observation $q(h_{t}|h_{t-1},y_{t})$ and not only the dynamics of the latent process itself. Scharth and Kohn [24] suggest using efficient importance sampling [25] inside the PMCMC procedure. Guarniero et al. [26] use twisted representation of the model and use the look-ahead type of particle filtering to address the issue of high variance of the estimated likelihood. Johansen and Doucet [27] compare sequential importance resampling (SIR) with auxiliary particle filter and find that APF does not always outperform SIR. Often, the variance of the estimated likelihood is analyzed in the true value of the parameters, such as in [24]. However, when using particle Markov Chain Monte Carlo, it is also of interest whether the same conclusions hold in different points of the parameter space. In particular, we never start running the algorithm at the point of the true parameter values. This means that if the variance of the estimated likelihood is much larger in some areas of the parameter space, the convergence of the algorithm can be affected. Having insights into how the variance of the estimated likelihood is different in the parameter space can help to make a more efficient choice of the starting point for the algorithm.

We first review the sequential Monte Carlo methods for the estimation of the likelihood. After that, we discuss Metropolis-Hastings algorithm and how SMC and Metropolis-Hastings can be combined for Bayesian inference in general and stochastic volatility models in particular.

#### 3.2.1. Sequential Monte Carlo

Assume that we are in the framework with an observed time series process $y_{t}$ and a latent Markovian process $h_{t}$. Since we never observe the latent process, we need to infer it. The objective that can be achieved with *Sequential Monte Carlo* (SMC) is also known as *particle filtering*. The method operates in sequential manner with arriving observations $y_{t}$. The posterior distribution of the latent process can be computed sequentially

(18) $$p\left(h_{0:t}|y_{1:t}\right)=p\left(h_{0:t-1}|y_{0:t-1}\right)\frac{g\left(y_{1:t}|h_{0:t}\right)f\left(h_{t}|h_{t-1}\right)}{p(y_{t}|y_{t-1})}.$$

The denominator of Equation (18) is not analytically tractable, which can be also seen from Equation (12) earlier. SMC allows us to estimate the posterior distribution $p(h_{0:t}|y_{1:t})$ and additionally get the estimate of the likelihood

(19) $$\begin{array}{c} L\left(y_{1:T}\right)=\int p\left(y_{1:T},h_{1:T}\right)dh_{1:T}=\int g\left(y_{1:T}|h_{1:T}\right)p\left(h_{1:T}\right)dh_{1:T}\\ =\int g\left(y_{1}|h_{1}\right)p\left(h_{1}\right)\prod_{t=2}^{T}g\left(y_{t}|h_{t}\right)f\left(h_{t}|h_{t-1}\right)dh_{1}\cdots h_{T}. \end{array}$$

The basic procedure of particle filtering in this setting can be summarized by three crucial steps: prediction, updating, and resampling. The outline of a basic particle filter can be summarized in the following way.

- Initialization: given the prior distribution $\pi (\theta_{0})$, we draw *N* independent random samples ${\left\{h_{i}^{0}\right\}}_{i=1}^{N}$; these samples we call *particles*.
- Prediction: we sample particles according to the importance density
  (20) $$h_{t}^{\left(i\right)}∼q\left(h_{t}|h_{t-1}^{\left(i\right)},y_{t}\right).$$
- Updating: During updating, we assign a weight $w_{t}^{\left(i\right)}$ to every particle
  (21) $$w_{t}^{\left(i\right)}=\frac{p\left(y_{t}|h_{t}^{\left(i\right)}\right)f\left(h_{t}^{\left(i\right)}|h_{t-1}^{\left(i\right)}\right)}{p\left(y_{t}|y_{1:t-1}\right)q_{t}\left(h_{t}^{\left(i\right)}|h_{0:t-1}^{\left(i\right)}\right)}$$
  and normalize these weights to sum to 1. Every weight can be interpreted as our “confidence” about a particle.
- Resampling: resample the particles if the effective number of particles,
  (22) $$N_{eff}=\frac{1}{\sum_{i=1}^{N}{\left(\omega_{t}^{\left(i\right)}\right)}^{2}},$$
  is too low. In Equation (22), $\omega_{t}^{\left(i\right)}$ is the normalized weight of particle *i* at the time step *k*. The threshold for the resampling step is set depending on whether particle degeneracy is a problem. In general, we perform resampling when $N_{eff}<N/c$, where *c* is a constant.

The resampling step is performed to find the trade-off between two well-documented problems: particle *degeneracy* and particle *impoverishment* [28]. The former happens when the resampling step is ignored or is not performed frequently enough. In this case, one ends up with a particle set that has zero weights. The latter problem happens when the particle set is resampled too frequently; then, eventually one gets one particle with a large weight and hence the particle set lacks the diversity. The way to find the balance between these two problems is resampling when the efficient number of particles is smaller than a certain threshold.

In this paper, we consider two particle filters: bootstrap and auxiliary particle filters. A generic particle filter is presented in Algorithm A1 [28]. The bootstrap filter is a variation of a more general approach—sequential importance sampling (resampling). The distinction of the bootstrap filter is the proposal mechanism for the particles. In the bootstrap particle filter the proposals for the particles are made on the basis of the dynamics of the model $f(h_{t}|h_{t-1})$. If $q\left(h_{t}|y_{t},h_{t-1}\right)=f\left(h_{t}|h_{t-1}\right)$, then the term $\frac{f(h_{t}|h_{t-1})}{q(h_{t}|y_{t},h_{t-1})}$ is equal to 1. In the case of the auxiliary particle filter, we also incorporate the current observation into the proposal mechanism $q(h_{t}|h_{t-1},y_{t})$. Incorporating the current observation into the proposal for the particles in some cases allows us to reduce the variance of the estimated likelihood. In our case, there is no analytical expression for the proposal density. In the next subsection, we discuss how it can be approximated as proposed in [23].

#### 3.2.2. Auxiliary Particle Filter for SV Model

Incorporating knowledge of $y_{t}$ into proposals for particles $q(h_{t}|h_{t-1},y_{t})$ can help to reduce the variance of the estimated likelihood and improve the approximation of the filtering distribution $p(h_{t}|y_{1:t})$. Note, however, that it is not always the case as has been shown in [27]. Only in the case of linear Gaussian state-space models does the proposal density from Equation (A2) have an analytical expression. Hence, for the stochastic volatility models, this term must be approximated. Pitt and Shephard [23] propose using non-blind proposals for the next generation of particles by first expanding $\log g(y_{t+1}|h_{t+1})$ to a second-order term around $\mu_{t+1}^{k}$ via Taylor expansion

(23) $$\begin{array}{c} \log g\left(y_{t+1}|h_{t+1},\mu_{t+1}^{k}\right)=\log p{\left(y_{t+1}|\mu_{t+1}^{k}\right)}^{\prime }\times \frac{\partial \log p(y_{t+1}|\mu_{t+1}^{k})}{\partial h_{t+1}}+\\ \frac{1}{2}\times {\left(h_{t+1}-\mu_{t+1}^{k}\right)}^{\prime }\times \frac{\partial^{2}\log p\left(y_{t+1}|\mu_{t+1}^{k}\right)}{\partial h_{t+1}h_{t+1}^{\prime }}\times \left(h_{t+1}-\mu_{t+1}^{k}\right) \end{array}$$

For deriving the expression for $\log g(y_{t+1}|h_{t+1},\mu_{t+1}^{k})$, recall that $y_{t}∼N\left(0,\exp\left(h_{t}\right)\right)$ and hence

(24) $$\begin{array}{c} g\left(y_{t}|h_{t}\right)=\frac{1}{\sqrt{2\pi \exp(h_{t})}}\exp\left\{-\frac{y_{t}^{2}}{2\exp(h_{t})}\right\}=\\ \frac{1}{\sqrt{2\pi }}\exp\left\{-\frac{y_{t}^{2}}{\exp(h_{t})}-\frac{h_{t}}{2}\right\} \end{array}$$

and further note that $f\left(h_{t}|h_{t-1}\right)=N\left(\mu +\phi \left(h_{t-1}-\mu \right),\sigma_{\eta }^{2}\right)$; thus

(25) $$f\left(h_{t}|h_{t-1}\right)=\frac{1}{\sqrt{2\pi \sigma_{\eta }^{2}}}\exp\left\{\frac{{\left(h_{t}-\mu -\phi \left(h_{t-1}-\mu \right)\right)}^{2}}{2\sigma_{\eta }^{2}}\right\}.$$

It follows that the proposal for particles at time t+1 when taking into account the observation of the same period is

(26) $$q\left(h_{t+1}|h_{t}^{\left(k\right)},y_{t+1};\mu_{t+1}^{\left(k\right)}\right)=N\left(\mu_{t+1}^{\left(k\right)}+\frac{\sigma^{2}}{2}\left(\frac{y_{t}^{2}}{\beta^{2}}\exp\left(-\mu_{t+1}^{\left(k\right)}\right)-1\right),\sigma^{2}\right).$$

#### 3.2.3. Metropolis–Hastings

In this section, we consider the problem of sampling from the posterior distribution and a general algorithm to construct such a sampling scheme. With the Metropolis–Hastings algorithm, we sample from the posterior distribution by proposing a transition $\theta \to \theta^{*}$ with the density $q(\theta^{*}|\theta )$, which we accept with probability

(27) $$\alpha \left(\theta ,\theta^{*}\right)=\min\left\{1,\frac{\tilde{p}\left(\theta^{*}\right)}{\tilde{p}\left(\theta \right)}\frac{q(\theta |\theta^{*})}{q(\theta^{*}|\theta )}\right\},$$

where $\tilde{p}\left(\cdot \right)$ is a function proportional to our target distribution. A common choice for the proposal distribution is a random-walk, which we also use when applying PMCMC later in this paper, $q\left(\theta^{*}|\theta \right)=N\left(\theta^{*}|\theta ,\Sigma \right)$. The Metropolis–Hastings algorithm is one of the off-the-shelf MCMC methods in the statistical community. It is quite general and can be applied to various problems. The implementation of the Metropolis–Hastings algorithm requires specification of multiple quantities. We need to specify a conditional density $q(\theta^{*}|\theta )$ that is a proposal distribution, generally $q(\theta^{*}|\theta )$ should be such that we can easily simulate from it. In many applications, including ours, it is reasonable to take the Gaussian distribution as proposal distribution. In this case, it is also symmetric, meaning $q\left(\theta^{*}|\theta \right)=q\left(\theta |\theta^{*}\right)$. The Metropolis-Hastings iteration is outlined in the Algorithm 1.

In this algorithm, $\alpha (\theta ,\theta^{*})$ is the Metropolis–Hastings acceptance probability, where θ is the current state of the chain and $\theta^{*}$ is the candidate state of the parameter vector. Generally, in the simulations, it is desired to have around 25% of proposed candidate values accepted [29]. The idea is that when the proposal steps are too large (we make a proposal that is far away from the current state, θ, in the Markov chain), we do not explore local regions sufficiently well; moreover many of the candidates are then very likely to be rejected. When the proposal steps are very small, the acceptance rate will be very high, however, then we are not likely to leave regions of the local maximum or the convergence will happen very slowly.
**Algorithm 1** Metropolis-Hastings Algorithm.1:Given $\theta^{\left(t\right)}$,2:Generate $\theta_{t}^{*}∼q\left(\theta^{*}|\theta^{\left(t\right)}\right)$,3:Take          
$\theta^{\left(t+1\right)}=\left\{\begin{array}{c} \theta_{t}^{*},\;\;\;\mathrm{with}\;\mathrm{probability}\;\;\;\alpha \left(\theta^{\left(t\right)},\theta_{t}^{*}\right)\\ \theta^{\left(t\right)}\;\;\;\mathrm{with}\;\mathrm{probability}\;\;\;1-\alpha \left(\theta^{\left(t\right)},\theta_{t}^{*}\right), \end{array}\right.$where             
$\alpha \left(\theta ,\theta^{*}\right)=\min\left(1,\frac{\tilde{p}\left(\theta^{*}\right)}{\tilde{p}\left(\theta_{t}\right)}\frac{q(\theta |\theta^{*})}{q(\theta^{*}|\theta )}\right)$

The performance of Metropolis–Hastings depends on the choice of q(·) proposal distribution. In the simulation studies, we consider random-walk proposals of the form $\theta_{i+1}^{*}=\theta_{i}+\epsilon_{i}$, where *i* is iteration of the algorithm and $\epsilon_{i}$ is assumed to be Gaussian. More information on the theoretical properties of this algorithm can be found in [30].

#### 3.2.4. Particle Metropolis-Hastings

Particle Markov Chain Monte Carlo (PMCMC) methods were introduced in [4]. The basic idea is that MCMC methods, and in particular, Metropolis–Hastings algorithm, which is of interest to us, can be combined with Sequential Monte Carlo to make draws from the posterior distributions of the parameters. Algorithm 2 presents the particle Metropolis–Hastings algorithm. The difference from the standard Metropolis-Hastings is in the quantity ${\hat{p}}_{\theta^{*}}\left(y_{1:T}\right)$, which is the estimate of the likelihood obtained with a particle filter conditioning on the parameters vector θ. In this algorithm, $q(\theta \left(i-1\right)|\theta^{*})$ is the proposal distribution (which cancels out when it is symmetric), and π(·) is prior distribution.
**Algorithm 2** Particle Metropolis-Hastings.1:Initialize algorithm at i=0 and initialize parameters θ(0)2:Run an SMC algorithm targeting $p_{\theta^{\left(0\right)}}\left(h_{1:T}|y_{1:T}\right)$, sample $h_{1:T}^{\left(0\right)}∼{\hat{p}}_{\theta^{\left(0\right)}}\left(\cdot |y_{1:T}\right)$ and let ${\hat{p}}_{\theta }^{\left(0\right)}\left(y_{1:T}\right)$ denote the marginal likelihood estimate for the initialized parameters3:**for** i=1,⋯,M **do**4:    Generate $\theta^{*}∼q\left(\theta^{*}|\theta^{\left(i-1\right)}\right)$,5:    Run an SMC algorithm targeting $p_{\theta^{*}}\left(h_{1:T}|y_{1:T}\right)$, sample $h_{1:T}^{*}∼{\hat{p}}_{\theta^{*}}\left(\cdot |y_{1:T}\right)$ and let $\hat{p_{\theta^{*}}}\left(y_{1:T}\right)$ denote the marginal likelihood estimate for the proposed parameters $\theta^{*}$6:    With probability
(28)$$\min\left\{1,\frac{{\hat{p}}_{\theta^{*}}\left(y_{1:T}\right)}{{\hat{p}}_{\theta^{\left(i-1\right)}}\left(y_{1:T}\right)}\frac{\pi (\theta^{*})}{\pi (\theta^{\left(i-1\right)})}\frac{q(\theta^{\left(i-1\right)}|\theta^{*})}{q(\theta^{*}|\theta^{\left(i-1\right)})}\right\}$$7:    Set $\theta^{\left(i\right)}=\theta^{*}$, $h_{1:T}^{\left(i\right)}=h_{1:T}^{*}$ and ${\hat{p}}_{\theta^{\left(i\right)}}\left(y_{1:T}\right)={\hat{p}}_{\theta^{*}}\left(y_{1:T}\right)$;8:    Otherwise set $\theta^{\left(i\right)}=\theta^{\left(i-1\right)}$, $h_{1:T}^{\left(i\right)}=h_{1:T}^{\left(i-1\right)}$ and ${\hat{p}}_{\theta^{\left(i\right)}}\left(y_{1:T}\right)={\hat{p}}_{\theta^{\left(i-1\right)}}\left(y_{1:T}\right)$.9:**end for**

### 3.3. MCMC with Gradient Information

In this section, we discuss Riemann Manifold Langevin Hamiltonial Monte Carlo methods that are introduced in [5] and in particular can be applied to stochastic volatility models.

The method originates in physics statistical literature and provides a tool that allows one to make large transitions with high acceptance probability, something that standard methods such as Metropolis–Hastings fail to achieve. The idea of HMC is based on relation between differential geometry and statistical theory (MCMC in particular). Girolami and Calderhead [5] propose the Metropolis-adjusted Langevin algorithm and Hamiltonian Monte Carlo sampling algorithms that are defined on the Riemann manifold. Their methods allow us to overcome the problem of sampling from high-dimensional densities that may show strong correlation. We further provide the general background and summary of the algorithms together with the necessary quantities for their implementation in the case of stochastic volatility models. It is not our goal to provide theoretical foundations of these methods in this article. For deeper theoretical foundations, see [31,32,33].

In standard MCMC setting, one uses probability distribution to make a proposal for the next state of the Markov chain. Hamiltonian Monte Carlo methods exploit physical system dynamics to make proposals for the next state. It can improve the mixing drastically and result in a more efficient algorithm. Especially since we are interested in multivariate modeling, a more efficient exploration of the posterior distribution is of interest. Once the dimension of the model grows with standard random walk, it is very hard to make proposals that would be accepted frequently enough and result in a good mixing Markov chain. We first introduce some basic ideas on which Hamiltonian Monte Carlo method is built; for an extensive introduction, we refer to [33].

#### 3.3.1. Metropolis-Adjusted Langevin Algorithm

Previously we have discussed the Metropolis-Hastings algorithm. The idea of the Metropolis-Hastings algorithm is to make a new proposal $\theta^{*}$ using random walk. Then this proposal is accepted with probability.

(29) $$\alpha \left(\theta ,\theta^{*}\right)=\min\left\{1,\frac{\tilde{p}\left(\theta^{*}\right)}{\tilde{p}\left(\theta \right)}\frac{q(\theta |\theta^{*})}{q(\theta^{*}|\theta )}\right\}.$$

Although this algorithm benefits from desirable theoretical guarantees, the random walk proposal is not efficient, especially when the number of parameters in the model becomes large. Metropolis-adjusted Langevin algorithm (MALA), originally proposed in [34], is designed to solve the same problem—sample from the target distribution. The big advantage of MALA in comparison to Metropolis–Hastings is the construction for the proposal of the candidate parameter $\theta^{*}$. The proposal mechanism for MALA originates from the stochastic differential equation based on Langevin diffusion; the proposal mechanism reads

(30) $$\theta^{*}=\theta^{n}+\epsilon^{2}\nabla_{\theta }L\left(\theta^{n}\right)/2+\epsilon z^{n},$$

where we define $L\left(\theta^{n}\right)=\log\left(p\left(\theta \right)\right)$ and z∼N(z|0,I) and ϵ—integration step size. Convergence for this proposal is not guaranteed unless we employ a Metropolis acceptance probability after every integration step. For convenience, let us define

(31) $$\mu \left(\theta^{n},\epsilon \right)=\theta^{n}+\frac{\epsilon^{2}}{2}\nabla_{\theta }L\left(\theta^{n}\right);$$

then the proposal density can be written as $q\left(\theta^{*}|\theta^{n}\right)=N\left(\theta^{*}|\mu \left(\theta^{n},\epsilon \right),\epsilon^{2}I\right).$ The standard acceptance probability follows

(32) $$\min\{1,p\left(\theta^{*}\right)q\left(\theta^{n}|\theta^{*}\right)/p\left(\theta^{n}\right)q\left(\theta^{*}|\theta^{n}\right)\}.$$

The type of proposal in Equation (29) is inefficient for strongly correlated parameters θ. To solve this issue, one can use a preconditioning matrix M

(33) $$\theta^{*}=\theta^{n}+\epsilon^{2}M\nabla_{\theta }L\left(\theta^{n}\right)/2+\epsilon \sqrt{M}z^{n}.$$

Unfortunately there is no principled way to choose matrix M. As we will see later, HMC encounters the same problem. Generally, MALA iterates between two general steps. First, Langevin dynamics is used for the proposals, and it exploits the gradients of the target. Second, the proposals are accepted or rejected similarly to those of the Metropolis–Hastings algorithm.

#### 3.3.2. Hamiltonian Monte Carlo Algorithm

The HMC algorithm [31] also uses gradient information for constructing the proposal of the parameters in the MCMC scheme. In particular, it exploits the ideas from simulating the behavior of the physical systems. Similarly to describing the behavior of the physical system, HMC performs sampling by exploiting *Hamiltonian dynamics*. A conceptual introduction to this class of methods and its relationship to differential geometry can be found in [33]. In this section, we discuss the general idea behind the algorithm without performing detailed derivations. We focus on the final proposal machinery that can be used in practice and investigate which quantities need to be manually computed before implementing the algorithm and which variables need to be calibrated for the successful performance of the algorithm. First, let us consider a general set-up. In Hamiltonian Monte Carlo, we consider a Hamiltonian function

(34) $$H\left(\theta ,p\right)=-\log p\left(\theta \right)+\frac{1}{2}{\log\{\left(2\pi \right)}^{D}\left|M|\right\}+\frac{1}{2}p^{T}M^{-1}p,$$

which consists of potential energy in the system E(θ)=−L(θ) and kinetic energy $K\left(p\right)=\frac{1}{2}{\log\{\left(2\pi \right)}^{D}\left|M|\right\}+\frac{1}{2}p^{T}M^{-1}p$; variables p are called momentum variables. The dynamics of the system then evolves according to Hamiltonian equations

(35) $$\frac{d\theta }{d\tau }=\frac{\partial H}{\partial p}=M^{-1}p,$$

(36) $$\frac{dp}{d\tau }=-\frac{\partial H}{\partial \theta }=\nabla_{\theta }L\left(\theta \right),$$

where by τ in physical interpretation of the system we denote continuous time. Practical implementation requires discretization, and the commonly used scheme for this purpose is the leapfrog discretezation:

(37) $$p\left(\tau +\epsilon /2\right)=p\left(\tau \right)+\epsilon \nabla_{\theta }L\left\{\theta \left(\tau \right)\right\}/2,$$

(38) $$\theta \left(\tau +\epsilon \right)=\theta \left(\tau \right)+\epsilon M^{-1}p\left(\tau +\epsilon /2\right),$$

(39) $$p\left(\tau +\epsilon \right)=p\left(\tau +\epsilon /2\right)+\epsilon \nabla_{\theta }L\left\{\theta \left(\tau +\epsilon \right)\right\}/2.$$

This scheme does not sample from the target distribution and to correct for that, implementation of Metropolis acceptance probability is necessary. For a proposal $\left(\theta ,p\right)\to \left(\theta^{*},p^{*}\right)$, acceptance probability in this algorithm is defined as

$$\min\{1,\exp\left\{-H\left(\theta^{*},p^{*}\right)+H\left(\theta ,p\right)\right\}.$$

Thus, HMC iterates between updating momentum variables, proposal for the parameter values, additional update to the momentum variables, and then an acceptance/rejection step. The Gibbs sampler provides a good understanding for the system evolution in this algorithm:

(40) $$p^{n+1}|\theta^{n}∼p\left(p^{n+1}|\theta^{n}\right)=p\left(p^{n+1}\right)=N\left(p^{n+1}|0,M\right),$$

(41) $$\theta^{n+1}|p^{n+1}∼p\left(\theta^{n+1}|p^{n+1}\right)$$

Similarly to MALA, the choice of matrix M is crucial for good performance of HMC. While the choice of the step size and the leapfrog steps can be tuned relatively easily by considering acceptance rate, the choice of the matrix M is challenging, and there is no principled way to define it. Leapfrog step and step size proposal are two variables that need to be calibrated when implementing HMC. Usually, different combinations of these two variables are considered, and the combination leading to the highest acceptance rate is picked.

### 3.4. Riemann Manifold Hamiltonian Monte Carlo

The further improvement of HMC and MALA can done by defining the algorithms on Riemann manifold instead of Euclidean space. Proposals guided by Riemann metric instead of Euclidean distance have the potential to explore parameter space more efficiently, especially in the cases when the target density is high-dimensional or exhibits strong correlation [5]. The method originally proposed in [5] and multiple algorithms were compared in the paper: MALA, MMALA, HMC, and RMHMC. For detailed comparison between these methods, we refer to [5], while in our simulation studies, we will focus on comparing RMHMC and particle Metropolis–Hastings for the estimation of parameters in stochastic volatility models.

Girolami and Calderhead [5] define HMC methods in the form of Riemann manifold, and this can be seen as generalization of HMC. The Hamiltonian on the Riemann manifold is defined as follows

(42) $$H\left(\theta ,p\right)=-\log p\left(\theta \right)+\frac{1}{2}\log\left({\left(2\pi \right)}^{n}|G\left(\theta \right)|\right)+\frac{1}{2}p^{T}G\left(\theta \right)p$$

with exp(−H(θ,p))=p(θ,p)=p(θ)p(p∣θ) and the marginal target density

(43) $$\begin{array}{c} p\left(\theta \right)\propto \int \exp\left(-H\left(\theta ,p\right)\right)dp=\frac{\exp\{\log p(\theta )\}}{\sqrt{2\pi^{n}|G\left(\theta \right)|}}\int \exp\left\{-\frac{1}{2}p^{T}G{\left(\theta \right)}^{-1}p\right\}dp\\ =\exp(\log p(\theta )). \end{array}$$

The general idea behind the updates in RMHMC is similar to that of HMC, and the updates for the momentum variables and parameters of the model are defined in Equations (44)–(46).

(44) $$p\left(\tau +\frac{\epsilon }{2}\right)=p\left(\tau \right)-\frac{\epsilon }{2}\nabla_{\theta }H\left\{\theta \left(\tau \right),p\left(\tau +\frac{\epsilon }{2}\right)\right\},$$

(45) $$\theta \left(\tau +\epsilon \right)=\theta \left(\tau \right)+\epsilon /2\left[\nabla_{p}H\left\{\theta \left(\tau \right),p\left(\tau +\frac{\epsilon }{2}\right)\right\}+\nabla_{p}H\left\{\theta \left(\tau +\epsilon \right),p\left(\tau +\frac{\epsilon }{2}\right)\right\}\right],$$

(46) $$p\left(\tau +\epsilon \right)=p\left(\tau +\frac{\epsilon }{2}\right)-\frac{\epsilon }{2}\nabla_{\theta }H\left\{\theta \left(\tau +\epsilon \right),p\left(\tau +\frac{\epsilon }{2}\right)\right\}$$

Therefore, as in standard HMC algorithm, we iterate between half-step update of the momentum variables, and then we update position variables, and we finish iteration with additional half-step update of the momentum variables and Metropolis acceptance/rejection step with the probability

$$\min\{1,\exp\left\{-H\left(\theta^{*},p^{*}\right)+H\left(\theta^{n},p^{n+1}\right)\right\}\}.$$

Similarly to HMC, RMHMC can be viewed as a Gibbs sampling scheme

(47) $$p^{n+1}|\theta^{n}∼p\left(p^{n+1}|\theta^{n}\right)=N\left\{p^{n+1}|0,G\left(\theta^{n}\right)\right\},$$

(48) $$\theta^{n+1}|p^{n+1}∼p\left(\theta^{n+1}|p^{n+1}\right).$$

Recall that in the case of MALA and HMC, matrix M has to be chosen manually and there is no principled way to choose it. In RMHMC, matrix G(θ) is defined at each step by underlying geometry; see for more details [5]. Below we discuss quantities that need to be computed for the implementation of RMHMC in the case of stochastic volatility model and in particular G(θ).

Recall stochastic volatility model parametrized through β

(49) $$y_{t}=\beta \exp\left(h_{t}/2\right)\epsilon_{t},$$

(50) $$h_{t+1}=\phi h_{t}+\eta_{t+1},$$

$\epsilon_{t}∼N\left(0,1\right)$, $\eta_{t}∼N\left(0,\sigma^{2}\right)$, with $h_{1}∼N\left(0,\sigma^{2}/\left(1-\phi^{2}\right)\right)$.

The joint likelihood of the model is

(51) $$p\left(y,h,\beta ,\phi ,\sigma \right)=\prod_{t=1}^{T}p\left(y_{t}|h_{t},\beta \right)\prod_{t=2}^{T}p\left(h_{t}|h_{t-1},\phi ,\sigma \right)\pi \left(\beta \right)\pi \left(\phi \right)\pi \left(\sigma \right)$$

The prior distributions are chosen as follows

(52) $$\beta \propto \exp\left(\beta \right),\;\sigma^{2}∼Inv-\chi^{2}\left(10,0.05\right),\;\left(\phi +1\right)/2∼Beta\left(20,1.5\right).$$

Further, following [5], we write the partial derivatives for L=p(y,h∣β,ϕ,σ)

(53) $$\frac{\partial L}{\partial \beta }=-\frac{T}{\beta }+\sum_{t=1}^{T}\frac{y^{2}}{\beta^{3}\exp\left(h_{t}\right)},$$

(54) $$\frac{\partial L}{\partial \phi }=-\frac{\phi }{\left(1-\phi^{2}\right)}+\frac{\phi h_{1}^{2}}{\sigma^{2}}+\sum_{t=2}^{T}\frac{h_{t-1}\left(h_{t}-\phi h_{t-1}\right)}{\sigma^{2}},$$

(55) $$\frac{\partial L}{\partial \sigma }=-\frac{T}{\sigma }+\frac{h_{1}^{2}\left(1-\phi^{2}\right)}{\sigma^{3}}+\sum_{t=2}^{T}\frac{{\left(h_{t}-\phi h_{t-1}\right)}^{2}}{\sigma^{3}}.$$

To implement the algorithms, we require the expressions for the individual components of the metric tensor for the likelihood. Following [5], the expressions are

(56) $$\begin{array}{c} E\left\{\frac{\partial L}{\partial \beta }\frac{\partial L}{\partial \beta }\right\}=\frac{2T}{\beta^{2}},\;E\left\{\frac{\partial L}{\partial \sigma }\frac{\partial L}{\partial \sigma }\right\}=\frac{2T}{\sigma^{2}},\;E\left\{\frac{\partial L}{\partial \beta }\frac{\partial L}{\partial \sigma }\right\}=E\left\{\frac{\partial L}{\partial \beta }\frac{\partial L}{\partial \phi }\right\}=0, \end{array}$$

(57) $$\begin{array}{c} E\left\{\frac{\partial L}{\partial \sigma }\frac{\partial L}{\partial \phi }\right\}=\frac{2\phi }{\sigma^{3}\left(1-\phi^{2}\right)},\;E\left\{\frac{\partial L}{\partial \phi }\frac{\partial L}{\partial \phi }\right\}=\frac{2\phi^{2}}{{\left(1-\phi^{2}\right)}^{2}}+\frac{T-1}{1-\phi^{2}}. \end{array}$$

Furthermore, the expressions for the metric tensor for the likelihood and its partial derivatives follow

(58) $$G\left(\phi ,\sigma ,\beta \right)=\left[\begin{array}{ccc} \frac{2T}{\beta^{2}} & 0 & 0\\ 0 & \frac{2T}{\sigma^{2}} & \frac{2\phi }{\sigma^{3}\left(1-\phi^{2}\right)}\\ 0 & \frac{2\phi }{\sigma^{3}\left(1-\phi^{2}\right)} & \frac{2\phi^{2}}{{\left(1-\phi^{2}\right)}^{2}}+\frac{T-1}{1-\phi^{2}} \end{array}\right],$$

(59) $$\frac{\partial G}{\partial \beta }=\left[\begin{array}{ccc} -\frac{4T}{\beta^{3}} & 0 & 0\\ 0 & 0 & 0\\ 0 & 0 & 0 \end{array}\right],$$

(60) $$\frac{\partial G}{\partial \sigma }=\left[\begin{array}{ccc} 0 & 0 & 0\\ 0 & -\frac{4T}{\sigma^{3}} & -\frac{6\phi }{\sigma^{4}\left(1-\phi^{2}\right)}\\ 0 & -\frac{6\phi }{\sigma^{4}\left(1-\phi^{2}\right)} & 0 \end{array}\right],$$

(61) $$\frac{\partial G}{\partial \phi }=\left[\begin{array}{ccc} 0 & 0 & 0\\ 0 & 0 & \frac{2}{\sigma^{3}\left(1-\phi^{2}\right)}+\frac{4\phi^{2}}{\sigma^{3}{\left(1-\phi^{2}\right)}^{2}}\\ 0 & \frac{2}{\sigma^{3}\left(1-\phi^{2}\right)}+\frac{4\phi^{2}}{\sigma^{3}{\left(1-\phi^{2}\right)}^{2}} & \frac{2\phi (1+T)}{{\left(1-\phi^{2}\right)}^{2}}+\frac{6\phi^{3}}{{\left(1-\phi^{2}\right)}^{3}} \end{array}\right].$$

The proposal machinery in RMHMC provides advantages for exploring parameter space efficiently. However, it is not easily adaptable for different model specifications, especially when increasing the model’s dimensionality, as we discussed in Section 1. In particular, although matrix G can be computed in the multivariate model specified in Equations (12) and (13) exactly, it scales quadratically with the number of parameters. This might be one of the reasons why the method has not been used on multivariate stochastic volatility models we introduced in Section 1. However, probabilistic programming languages [35,36] and automatic differentiation possibilities developed in recent years allow the efficient and adaptable implementation of these algorithms in practice.

### 3.5. Integrated Nested Laplace Approximation

Integrated Nested Laplace Approximation was introduced in [7]. The method is based on the nested version of the classical Laplace approximation and was introduced for latent Gaussian models (LGMs). It became a popular approach in Bayesian inference due to its good performance in the variety of models in the class of LGMs and its computational advantages over other methods in Bayesian literature. The computational appeal of this method comes from the possibility of exploiting sparse matrix computations when evaluating certain approximations. INLA has found its applications in many fields in the models where high-dimensional problems arise. Stochastic volatility models have been analyzed using INLA in [37,38]. Bivariate stochastic volatility model has been considered in [39], where the authors present and solve some issues that arise in using INLA in the multivariate case of the model. One of the conclusions of this study was that INLA loses its computational advantage with increased dimensionality of the stochastic volatility model. We further discuss the details of the method and the implementation shortcomings in a multivariate case and present the reader with a simulation study that illustrates the discussed approach’s performance.

Stochastic volatility model can be written in the form of LGMs

(62) $$y|h,\theta_{1}∼\prod_{i\in I}\pi \left(y_{i}|h_{i},\theta_{1}\right),$$

(63) $$h|\theta_{2}∼N\left(\mu \left(\theta_{2}\right),Q^{-1}\left(\theta_{2}\right)\right).$$

As before, $y_{t}$ is the data that we observe and $h_{t}$ is the latent volatility process, and we are interested in the posterior distribution of the parameters of the model θ and the latent process given the data

(64) $$p\left(h,\theta |y\right)\propto p\left(\theta \right)p\left(h|\theta \right)\prod_{t=1}^{T}p\left(y|h_{t},\theta \right).$$

The outline of the INLA approach can be summarized in the following steps [7,37]

(1) Build an approximation p(θ∣y)
(2) Build an approximation to $p(h_{t}|\theta ,y)$
(3) Compute an approximation to $p(h_{t}|y)$ using the approximations from steps 1 and 2.

The first approximation p(θ∣y) relies on the Gaussian approximation of the form

(65) $$p\left(x|y,\theta \right)\propto \exp\left\{-\frac{1}{2}x^{T}Qx+\sum g_{t}\left(h_{t}\right)\right\},$$

where x=(μ,h), $g_{t}\left(h_{t}\right)=\log p\left(y_{t}|h_{t},\theta \right)$. By matching the mode and curvature in the mode, we obtain the Gaussian approximation for our model

(66) $${\tilde{p}}_{G}\left(x|y,\theta \right)=K_{1}\exp\left\{-\frac{1}{2}{\left(x-\mu \right)}^{T}\left(Q+diag\left(c\right)\right)\left(x-m\right)\right\},$$

where $K_{1}$ is a normalizing constant, *m* is the modal value of p(x∣y,θ), the vector *c* contains the second order terms in the Taylor expansion of $\sum g_{t}\left(h_{t}\right)$ at the modal value m, and Q is the precision matrix that has the form

(67) $$Q=\left[\begin{array}{ccccc} 1 & -\phi & \; & & \\ -\phi & 1+\phi^{2} & -\phi & \; & \\ \; & \ddots & \ddots & \ddots & \\ \; & \; & -\phi & 1+\phi^{2} & -\phi \\ \; & \; & & -\phi & 1 \end{array}\right].$$

The sparsity of the precision matrix above allows one to exploit efficient sparse matrix computational methods and thus gain computational speed. Note that in the multivariate case, this advantage disappears since the matrix Q is not sparse anymore.

When it comes to the estimation of stochastic volatility models, approximation of the marginals $p(h_{t}|\theta ,y)$ is always the most challenging task. The solution that is proposed in [7] is (simplified) Laplace approximation of the form

(68) $$\log{\tilde{p}}_{SLA}\left(x_{t}|\theta ,y\right)=const-\frac{1}{2}x_{t}^{2}+\gamma_{t}^{\left(1\right)}\left(\theta \right)x_{t}+\frac{1}{6}x_{t}^{3}\gamma_{t}^{3}\left(\theta \right)+\cdots ,$$

where $\gamma_{t}^{\left(1\right)}$ and $\gamma_{t}^{\left(3\right)}$ are the terms in the Taylor expansion. The final step of the method is to approximate $p(x_{t}|y)$ with the numerical integration scheme

(69) $$\tilde{p}\left(x_{t}|y\right)=\sum_{k}\tilde{p}\left(x_{t}|\theta^{k},y\right)\tilde{p}\left(\theta^{k}|y\right){∆}_{k},$$

for some $\theta^{k}$ of θ, where $\theta^{k}$ is selected by creating a grid of points that covers the area of high density for $\tilde{p}\left(\theta |y\right)$. For  more details on implementation of the simplified Laplace approximation and the selection of grid of points for $\theta^{k}$, see [7,37].

### 3.6. Fixed-Form Variational Bayes

In this section, we discuss how the posterior distribution can be approximated using the fixed-form variational Bayes method proposed in [6]. The general idea of fixed-form variational inference consists in assuming a certain factorization of the prior distribution, which naturally leads to the factorized structure of the posterior. The factorizing distributions of the posterior are then assumed to come from a certain parametric family of distributions (for example, exponential) and instead of a sampling task, as in the previous section, we would perform the optimization task of minimizing the distance between the approximating distribution and the unknown posterior distribution.

As before, assume we observe a process ${\left\{y_{t}\right\}}_{t=1}^{T}$ that is driven by an unobservable or latent process ${\left\{h_{t}\right\}}_{t=1}^{T}$. Recall that Bayes’ rule gives us the posterior distribution of the parameters of the system

(70) p(h∣y)∝g(y∣h)π(h).

In the Bayesian framework, we formulate our prior beliefs, which we update once we acquire more data. In general, Variational Bayes methods focus on approximating the posterior distribution p(h∣y) with some distribution q(h∣y). Further, it is common to choose blocks of the parameters and impose independence for these blocks

(71) $$p\left(h|y\right)\approx q\left(h|y\right)=q\left(h_{1}|y\right)q\left(h_{2}|y\right).$$

By construction, the posterior of the blocks of the parameters is independent. In the literature, this is referred to as *the mean-field assumption*. To find the optimal approximation, we minimize the Kullback–Leibler (KL) divergence from q(h∣y) to p(h∣y)

(72) $$\tilde{p}\left(h|y\right)=\underset{q\left(h_{1}|\cdot \right)q\left(h_{2}|\cdot \right)}{arg min}KL\left(q\left(h_{1}|y\right)q\left(h_{2}|y\right)||p\left(h|y\right)\right).$$

Distributional approximation can be viewed as an optimization problem; i.e., an optimal distribution has to be chosen from the space of all possible distributions, and the KL divergence is chosen as a loss function [40]. Salimans et al. [6] propose a specific approach to the minimization problem of KL divergence, which is based on the similarities between the optimal solution to the problem and linear regression. The general idea of the approach is summarized as comprising the following steps:

- initialize all the posterior approximations q(θ);
- iterate over the parameters updating every one of them given the others;
- repeat until convergence.

Consider the stochastic volatility model

(73) $$y_{t}=\beta \exp\left(h_{t}/2\right)\epsilon_{t}$$

(74) $$h_{t+1}=\phi h_{t}+\eta_{t+1},$$

with $h_{1}∼N\left(0,\sigma^{2}/\left(1-\phi^{2}\right)\right)$ and $\epsilon_{t}∼N\left(0,1\right)$, $\eta_{t}∼N\left(0,\sigma_{\eta }^{2}\right)$. We specify our a priori beliefs in the following manner

(75) $$p\left(\beta \right)\propto \beta^{-1}\;\left(\phi +1\right)/2∼Beta\left(20,1.5\right),\;\sigma^{2}∼IG\left(5,0.25\right).$$

To apply the Variational Bayes method, we need to specify the posterior approximations q(θ). It is convenient to assume a hierarchical structure of the prior, in which case it factorizes to

(76) $$p\left(\phi ,\sigma^{2},\beta ,f\right)=p\left(\phi \right)p\left(\sigma^{2}\right)p\left(f|\phi ,\sigma^{2}\right)p\left(y|f\right),$$

where $f=(\log\left(\beta \right),h^{\prime })$. The hierarchical structure of the prior leads to the following factorization of the posterior approximation

(77) $$q_{\xi }\left(\sigma_{\eta }^{2},f|f\right)=q_{\xi }\left(\sigma_{\eta }^{2}\right)q_{\xi }\left(f|\phi ,\sigma^{2}\right)=\frac{q_{\xi }\left(\sigma_{\eta }^{2}|\phi \right)p\left(f|\phi ,\sigma^{2}\right)q_{\xi }\left(y|f\right)}{q_{\xi }\left(y|\phi ,\sigma^{2}\right)}.$$

Thus, the posterior approximations can be chosen as follows

(78) $$q_{\xi }\left(\left(\phi +1\right)/2\right)=Beta\left(\xi_{1},\xi_{2}\right),$$

(79) $$q_{\xi }\left(\sigma^{2}|\phi \right)∼IG\left(\xi_{3},\xi_{4}+\xi_{5}\phi^{2}\right),$$

(80) $$q(\log\left(\beta \right),h|\phi ,\sigma^{2})=N\left(m,V\right),$$

where

$$V^{-1}=P\left(\phi ,\sigma^{2}\right)+\xi_{6},\;m=V^{-1}\xi_{7},$$

with $P(\phi ,\sigma^{2})$ precision matrix of $p(log\left(\beta \right),h|\phi ,\sigma^{2})$.

Once the posterior approximations are initialized, we proceed with the next step and iterate over the parameters. The parameters are updated in blocks that correspond to the factorization of the posterior approximations. First, we update the block for the persistence parameter in the latent process $q_{\xi }\left(\phi \right)$

(81) $$\phi^{*}=s_{1}\left(\xi ,z_{1}^{*}\right),\;\;\;\mathrm{with}\;\;\;s_{1}\left(\right)\;\;\;\mathrm{and}\;\;\;z_{1}^{*}\;\;\;\mathrm{such}\;\mathrm{that}\;\;\;\sigma^{2*}∼q_{\xi }\left(\sigma^{2}|\phi^{*}\right),$$

(82) $$\sigma^{2*}=s_{2}\left(\xi ,z_{2}^{*},\phi^{*}\right),\;\;\;\mathrm{with}\;\;\;s_{2}\left(\right)\;\;\;\mathrm{and}\;\;\;z_{2}^{*}\;\;\;\mathrm{such}\;\mathrm{that}\;\;\;\sigma^{2*}∼q_{\xi }\left(\sigma^{2}|\phi^{*}\right),$$

(83) $${\hat{C}}_{1}=\nabla_{\xi }\left[s_{1}\left(\xi ,z_{1}^{*}\right)\right]\nabla_{\phi }\left[T_{1}\left(\phi^{*}\right)\right],$$

(84) $${\hat{g}}_{1}\approx \nabla_{\xi }\left[s_{1}\left(\xi ,z_{1}^{*}\right)\right]\left\{\nabla_{\phi }\left[\log p\left(\phi^{*}\right)+\log q_{\xi }\left(y|\phi^{*},\sigma^{2*}\right)-\log q_{\xi }\left(\sigma^{2*}|\phi^{*}\right)\right]\right\}.$$

Second, we update the block for the variance of the latent process $q_{\xi }\left(\sigma^{2}|\phi \right)$

(85) $${\hat{C}}_{2}=\nabla_{\xi }\left[s_{2}\left(\xi ,z_{2}^{*},\phi^{*}\right)\right]\nabla_{\sigma^{2}}\left[T_{2}\left(\sigma^{2*}\right)\right]$$

(86) $${\hat{g}}_{2}\approx \nabla_{\xi }\left[s_{2}\left(\xi ,z_{2}^{2*},\phi^{*}\right)\right]\nabla_{\sigma^{2}}\left[\log p\left(\sigma^{2*}\right)+\log q_{\xi }\left(y|\phi^{*},\sigma^{2*}\right)\right],$$

where $T_{2}\left(\sigma^{2*}\right)$ are the sufficient statistics of $q_{\xi }\left(\sigma^{2}|\phi \right)$. The last update is the update of the likelihood approximation

(87) $$a_{t+1}=\left(1-\omega \right)a_{t}+\omega E_{q_{\xi }\left(f|\phi^{*},\sigma^{2*}\right)}\left[\nabla_{f}\log p\left(y|f\right)\right],$$

(88) $$z_{t+1}=\left(1-\omega \right)z_{t}+\omega E_{q_{\xi }\left(f|\phi^{*},\sigma^{2*}\right)}\left[f\right],$$

(89) $$\xi_{6,t+1}=\left(1-\omega \right)\xi_{6,t}-\omega E_{q_{\xi }\left(f|\phi^{*},\sigma^{2*}\right)}\left[\nabla_{f}\nabla_{f}\log p\left(y|f\right)\right],$$

(90) $$\xi_{7,t+1}=a_{t+1}+\xi_{6,t+1}z_{t+1}.$$

For more extensive derivations of the updates, we refer the reader to [6]. Further, one might wonder how the latent process is estimated in this procedure. Salimans et al. [6] propose using the Kalman filter to estimate the filtering distribution. Even though it is a valid approach that is also undertaken in quasi-maximum likelihood method [41], its weakness lies in the linearization of the observation equation which implies that the distribution of the noise process is not longer Gaussian.

## 4. Results

In this section, we present results for the comparison of the discussed methods. We compare two particle filters (bootstrap and auxiliary particle filters) on the basis of bias, variance and on the estimated effective number of particles. We choose the better performing procedure of the two for using in the particle Metropolis–Hastings algorithm. We compare particle Metropolis–Hastings (PMH), Riemann Manifold Hamiltonian Monte Carlo (RMHMC), integrated nested Laplace approximation (INLA), and fixed-form variational Bayes (VB) on the basis of how well the posterior distributions obtained with these methods capture the ground truth (e.g., true parameter values). The ability of the methods to recover ground truth is assessed based on five simulated data sets with different underlying parameters. We additionally provide effective sample sizes for the comparison of the sampling methods (PMH and RMHMC). For illustration purposes, we also provide comparison on two real-world data sets.

### 4.1. Variance of the Estimated Likelihood

**Figure 2:**
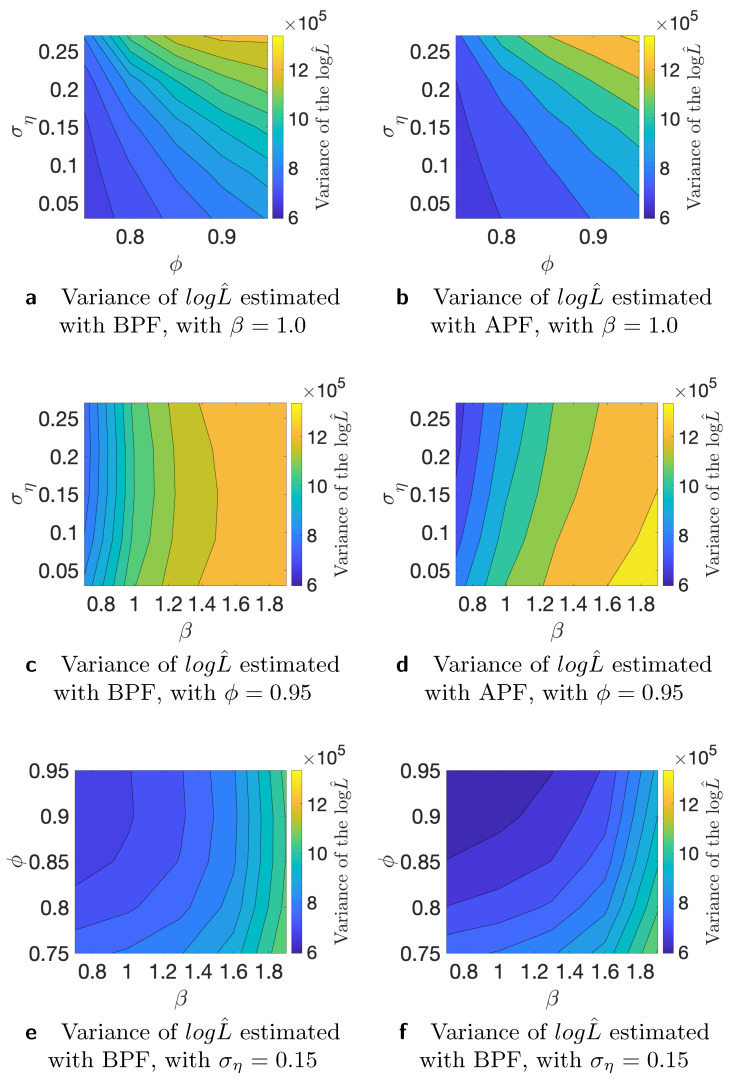
Variance of the estimated likelihood in different points of the parameter space for TS=2 from Table 2.

**Figure 3:**
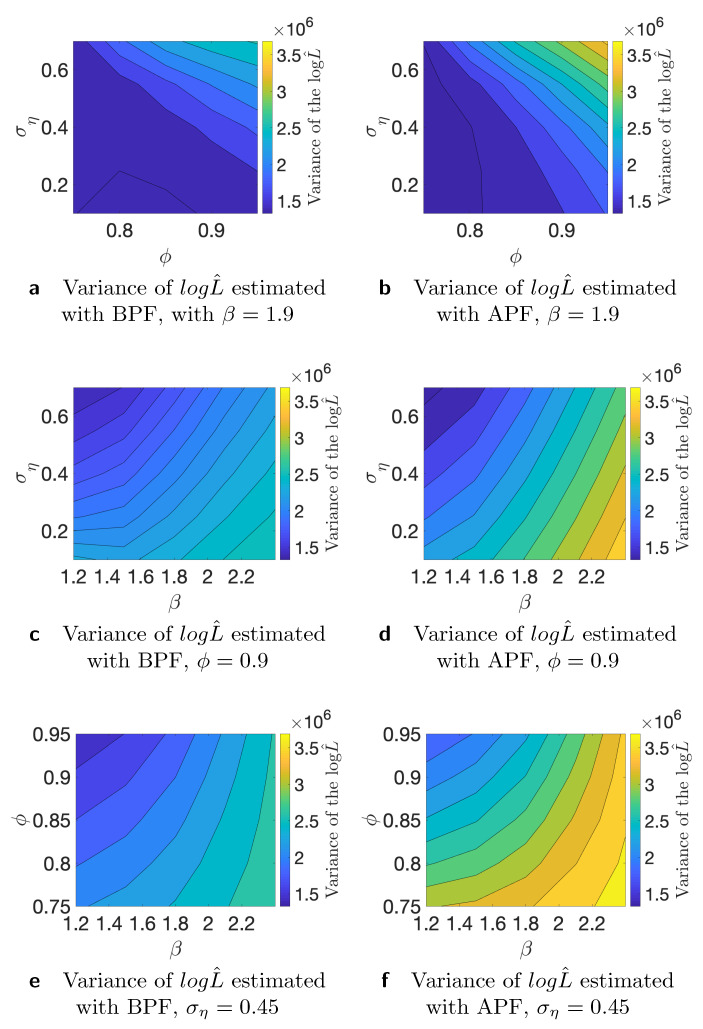
Variance of the estimated likelihood in different points of the parameter space for TS=3 from Table 2.

**Figure 4:**
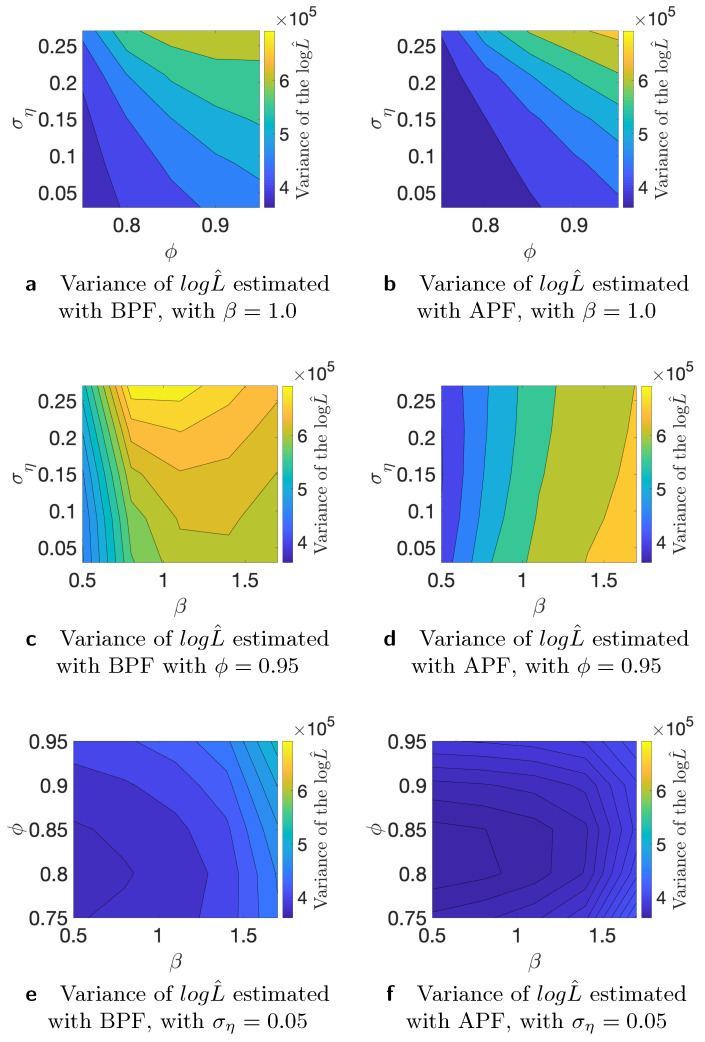
Variance of the estimated likelihood in different points of the parameter space for TS=4 from Table 2.

As we mentioned before, the marginal likelihood can be approximated sequentially through particle filtering. The marginal likelihood approximation of the parameters θ reads

(91) $$p\left(y_{1:T}|\theta \right)\approx \prod_{t}\hat{p}\left(y_{t}|y_{1:t-1},\theta \right),$$

where the right hand side is obtained by running particle filter presented in Algorithm A1. In practice, usually the log-likelihood

(92) $$\log p_{\theta }\left(y_{1:T}\right)=\log p_{\theta }\left(y_{1}\right)+\sum_{t=2}^{T}\log p_{\theta }\left(y_{t}|y_{1:t-1}\right)$$

is estimated for the purpose of numerical stability (as the product of small weights would lead to unstable results). The estimate of the log-likelihood is the by-product of the particle filtering, as it is the average over log-weights that are assigned to the particles at every time step. In this section, we compare the bootstrap (BPF) and auxiliary particle filters (APF) in terms of bias, variance, and number of effective particles. Both of them can be used for obtaining simulated likelihood estimates, which can be further used in the particle Metropolis–Hastings algorithm. We denote by $\hat{L}$ the estimate of the likelihood obtained with a particle filter. Then, the bias and the variance can be estimated as follows

(93) $$Bias=5000^{-1}\sum_{i=1}^{K}\sum_{j=1}^{M}\left(\log{\hat{L}}^{j}-\log\bar{L}\left(y^{i}\right)\right),$$

(94) $$Variance=5000^{-1}\sum_{i=1}^{K}\sum_{j=1}^{M}{\left(\log{\hat{L}}^{j}-\log\bar{L}\left(y^{i}\right)\right)}^{2},$$

where $y_{i}$ is the *i*-th time series, and logL is the “true” log-likelihood value. For the comparison, we use K=50 different time series generated from the stochastic volatility model and M=100 Monte Carlo iterations. We use N=100, N=1000, and *N* = 10,000 number of particles for this study. As the true value of the likelihood is not available, we substitute it with an estimate that is obtained with *N* = 1,000,000 number of particles. First, we conduct the analysis of the variance of the estimated likelihood in true parameter values as discussed in [24]. The authors of, ref. [27] showed theoretically that the asymptotic variance is not always smaller for the APF in comparison to the BPF. We run additional simulation studies to examine whether the variance of the estimated likelihood varies in the parameter space. Since we are interested in using the estimated likelihood in the Markov Chain Monte Carlo setting, it is relevant how the variance behaves in different points of the parameter space. If we start far away from the true value and the variance of the estimated likelihood is larger in that part of the parameter space, it can affect the convergence and calibration of the algorithm. Table 1 shows variance of the estimated likelihood for bootstrap and auxiliary particle filters. *N* indicates the number of the particles that we used for the estimation of the likelihood. It is clear that, on average, APF performs better in terms of the variance of the estimated likelihood. Table 2 indicates results for a similar experiment, but on the level of individual times series. We consider different data-generating processes and find that, in particular, higher variance of the latent volatility process is associated with higher variance of the estimated likelihood. Finally, in Figure 2, Figure 3 and Figure 4, we illustrate that the variance of the estimated likelihood changes depending on the location in the parameter space, and these changes can be specific to a data-generating process. These figures correspond to the experiments with time series 2, 3, and 4 from Table 2. The likelihood was estimated with N=1000 particles. We observe that the variance of the latent process has a strong effect on the landscape of the variance of the estimated likelihood in the parameter space. From Figure 4c,d, we see that the variance of the estimated likelihood obtained with the bootstrap particle filter appears to be more strongly affected by the location in the parameter space than the variance of the estimated likelihood obtained with the auxiliary particle filter. In Figure 3, we observe that the variance of the estimated likelihood is affected by the scale parameter β in the case of auxiliary particle filter, but not so much in the case of the bootstrap particle filter. Thus, initialization of PMCMC and the choice of number of particles should be considered with care for the optimal performance of the algorithm as the variance of the estimated likelihood can differ in the parameter space, and these changes can vary across different time series.

### 4.2. Particle Metropolis–Hastings and Riemann Manifold Hamiltonian Monte Carlo

**Figure 5:**
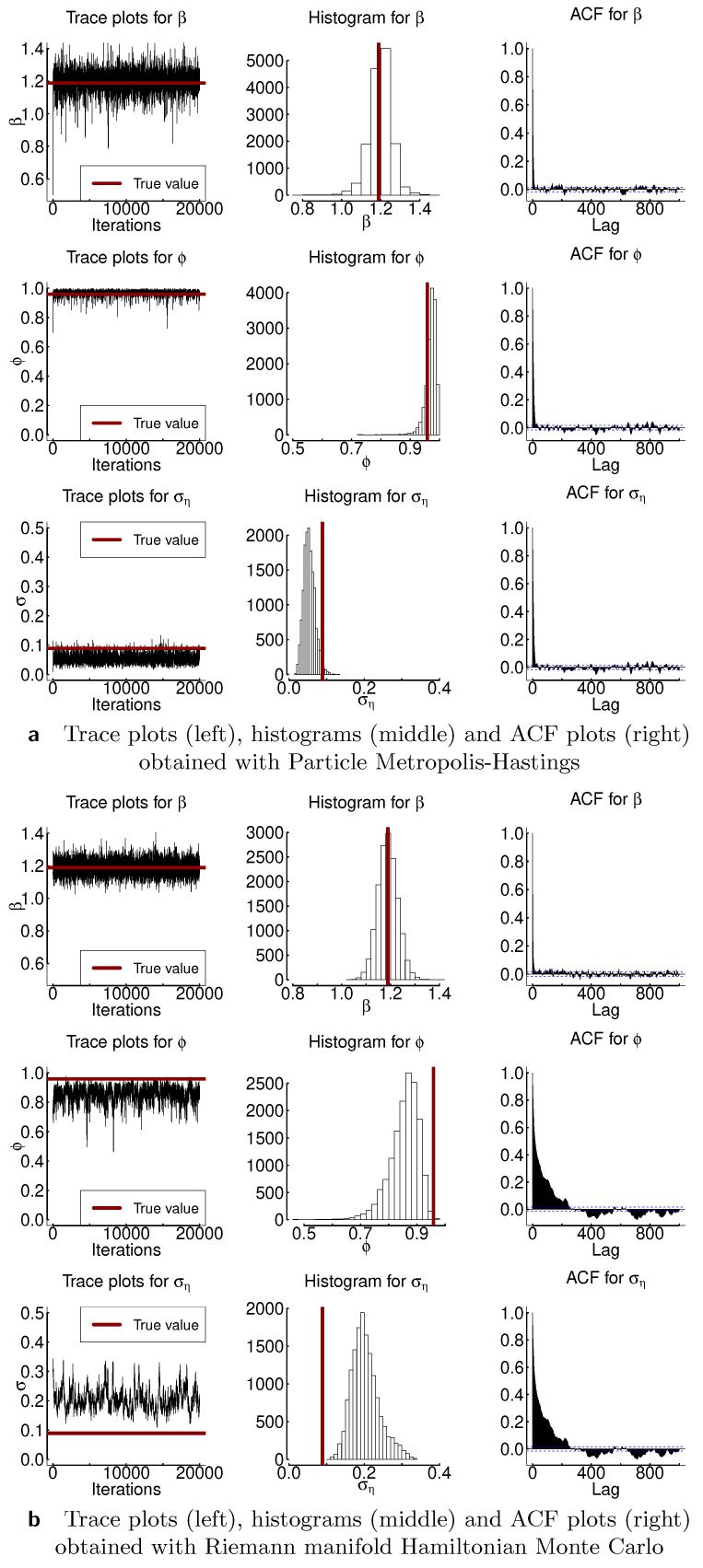
Results of the sampling from the posterior distribution with PMH and RMHMC for TS=2 from Table 2. The first column corresponds to the trace plots, the middle column to histograms obtained with the samples from the posterior distribution, and the last column corresponds to autocorrelation function for the samples.

**Figure A1:**
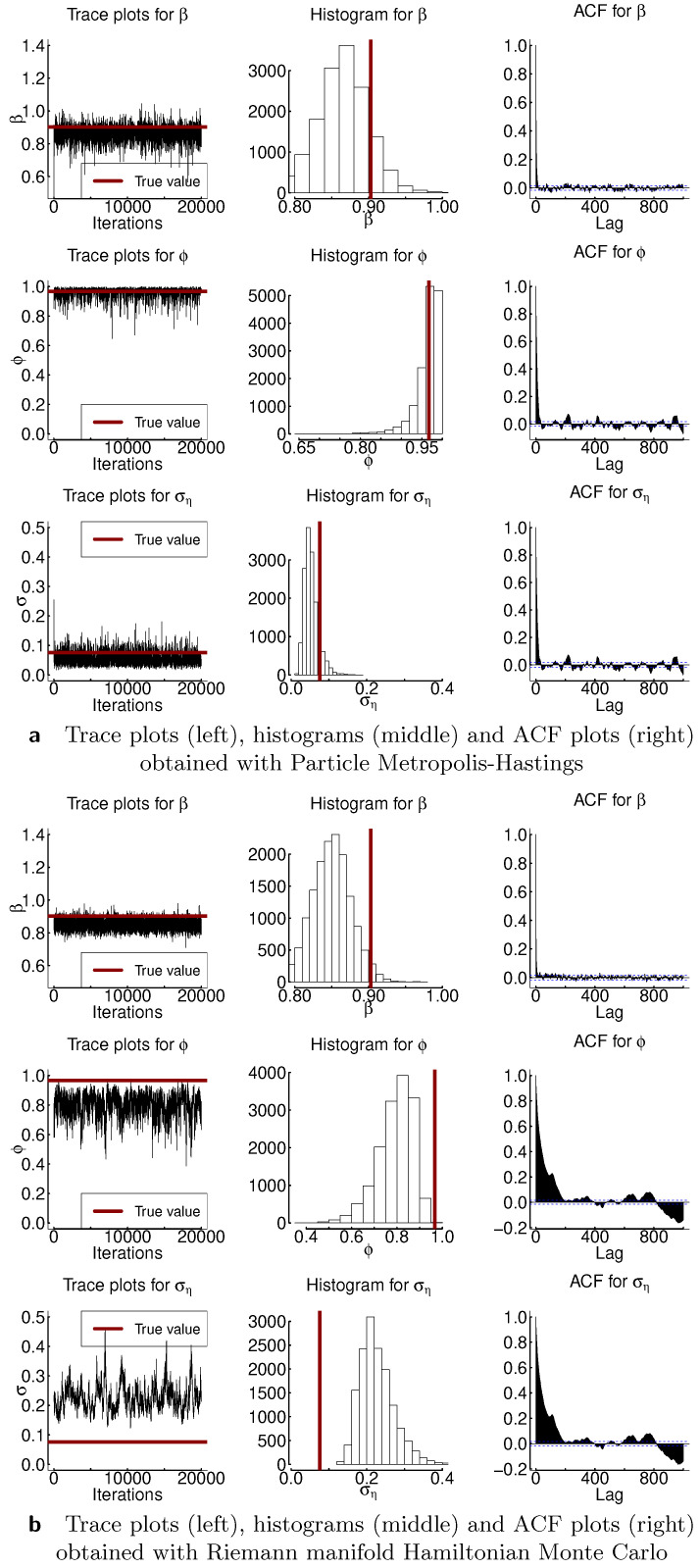
Results of the sampling from the posterior distribution with PMH and RMHMC for TS=4 from Table 2. The first column corresponds to the trace plots, the middle column to histograms obtained with the samples from the posterior distribution, and the last column to autocorrelation function for the samples.

**Figure A2:**
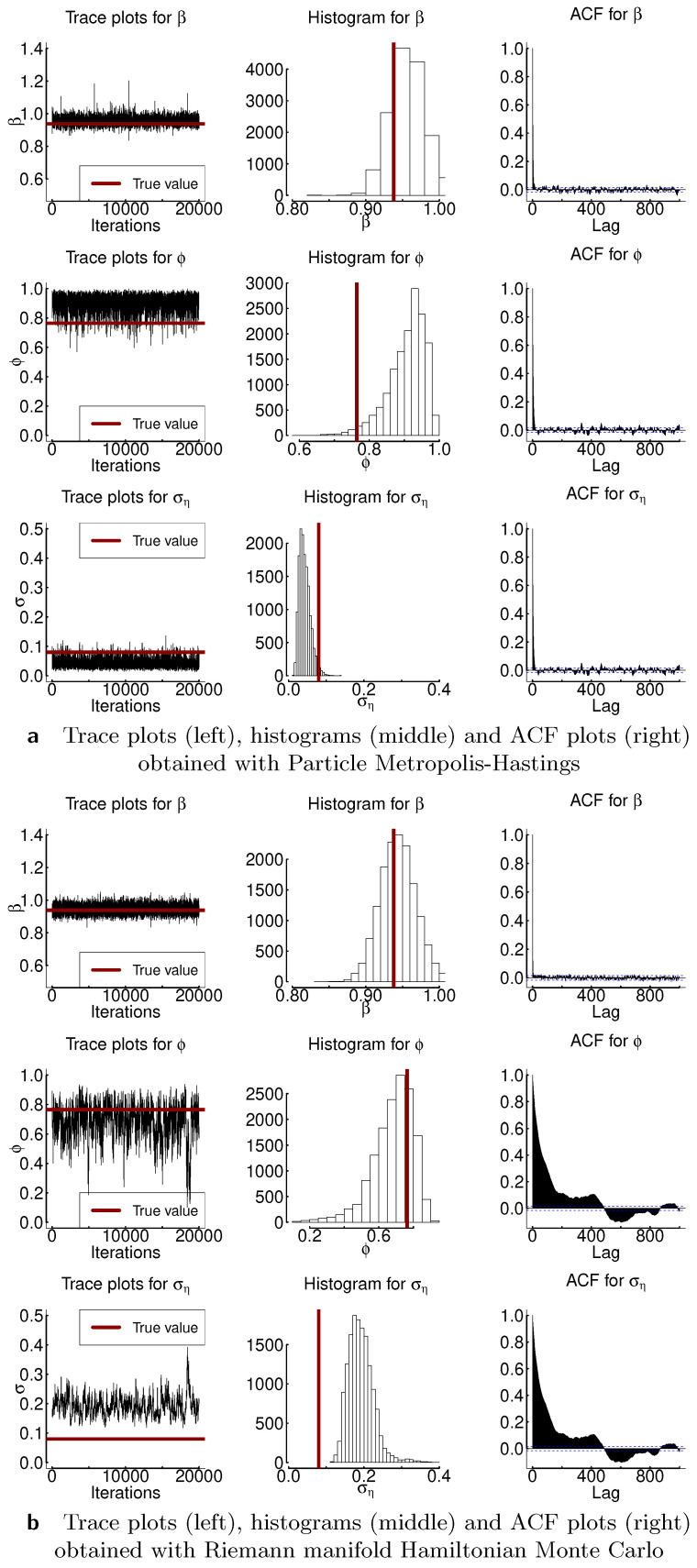
Results of the sampling from the posterior distribution with PMH and RMHMC for TS=5 from Table 2. The first column corresponds to the trace plots, the middle column to histograms obtained with the samples from the posterior distribution, and the last column to autocorrelation function for the samples.

**Figure A3:**
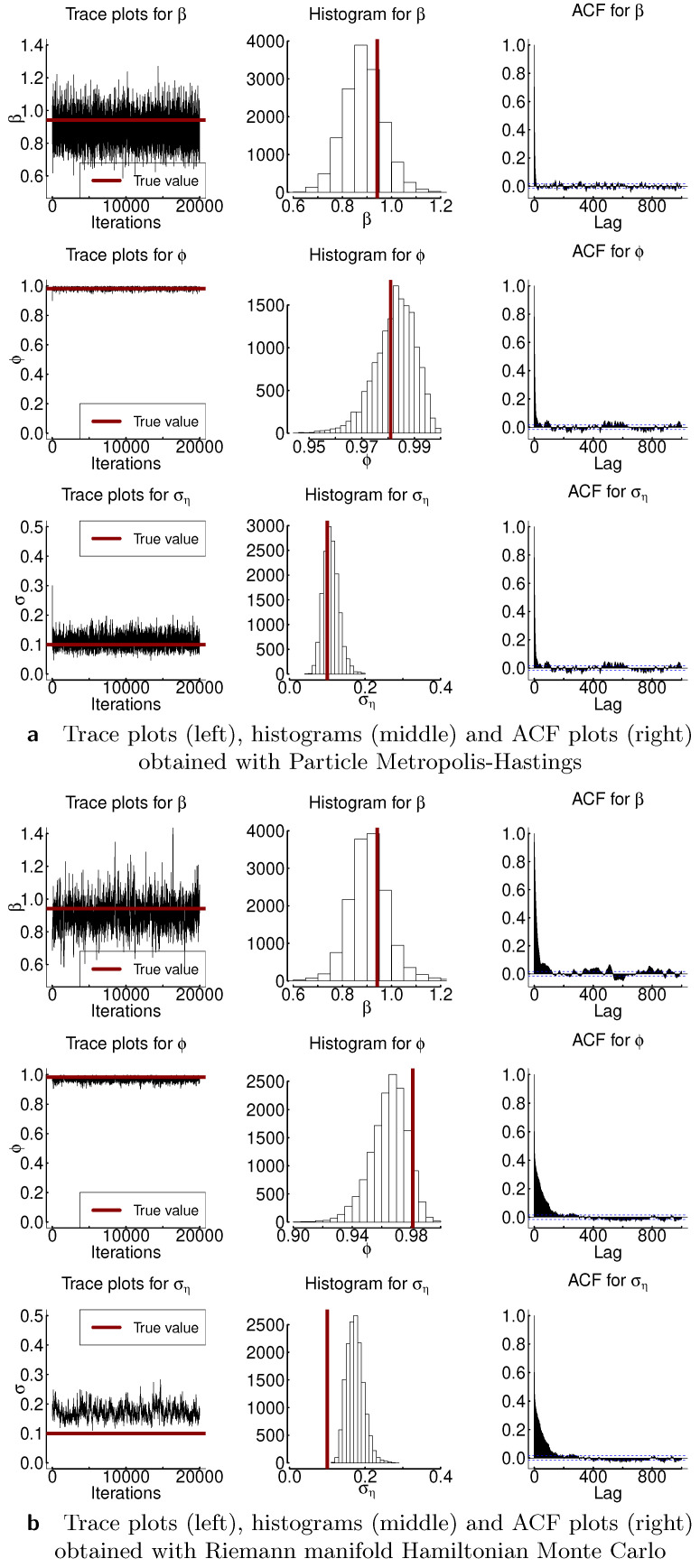
Results of the sampling from the posterior distribution with PMH and RMHMC for TS=9 from Table 2. The first column corresponds to the trace plots, the middle column to histograms obtained with the samples from the posterior distribution, and the last column to autocorrelation function for the samples.

**Figure A4:**
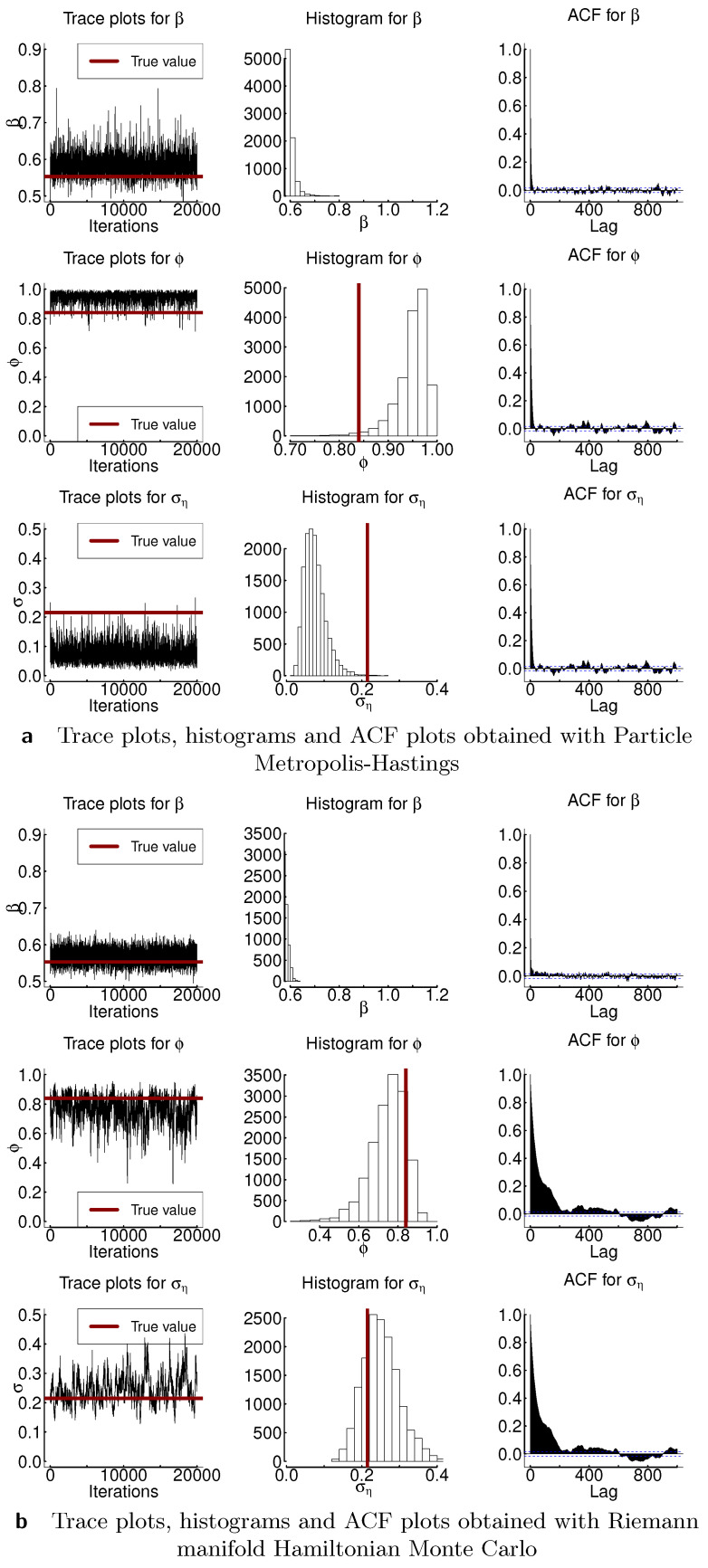
Results of the sampling from the posterior distribution with PMH and RMHMC for TS=10 from Table 2. The first column corresponds to the trace plots, the middle column to histograms obtained with the samples from the posterior distribution, and the last column to autocorrelation function for the samples.

In this section, we compare particle Metropolis–Hastings (PMH) and Riemann Manifold Hamiltonian Monte Carlo. We evaluate the algorithms based on how well they recover the true parameters of the model β, ϕ, and $\sigma_{\eta }$ and on the basis of the effective sample size. We obtained 20,000 samples and discarded the first 1000 as burn-in. Further, Figure 5 and Figure A1, Figure A2, Figure A3 and Figure A4 show results for both samplers: trace plots, histograms, and autocorrelation function are depicted. Table 3 presents the moments and highest posterior density intervals for the parameters of the model. The marginal likelihood in PMH was estimated with auxiliary particle filter as discussed in [23]. The Metropolis–Hastings part of the algorithm was calibrated to achieve 20–40% acceptance rate. RMHMC was implemented as in [5] with openly available implementation of the method by the authors. Both PMH and RMHMC require careful calibration of the step-size, and RMHMC additionally needs calibration of the number of the leapfrog steps; thus in Table A1, we additionally present results for the no-u-turn sampler (NUTS). NUTS is an extension of HMC algorithm that allows one to tune the algorithm automatically. From Figure 5 and Figure A1, Figure A2, Figure A3 and Figure A4, we observe that autocorrelation of the samples indicated in the third column of the plots decreases faster for PMH than for RMHMC, in particular for the parameters ϕ and $\sigma_{\eta }$. Effective sample size is similarly high for parameter β for both samplers. Effective sample size for the parameters ϕ and $\sigma_{\eta }$ is lower in the case of RMHMC. However, if we compute the ESS per second as presented in the last column of Table 3, this advantage disappears. This result is not surprising since PMH is the most computationally intensive procedure we are considering. Both the likelihood and the posterior use sequential sampling methods, which makes computations very demanding. Nevertheless, PMH allows us to recover the underlying parameters more accurately. In particular, in most of the presented examples, true variance of the latent volatility process lies inside the 95% highest posterior density interval for PMH. RMHMC tends to overestimate this parameter. As Table A1 indicates, the highest posterior density intervals obtained with NUTS are larger than those obtained with PMH and RMHMC. Number of gradient evaluations for RMHMC are 69718, 70042, 69802, 69801, and 70041 for Experiments 1–5, respectively.

### 4.3. Integrated Nested Laplace Approximation

We provide two simulation studies for the integrated nested Laplace approximation. First, we replicate and extend the simulation study provided in [38] by analyzing data-generating processes with different values of μ and $\sigma_{\eta }$ since the estimation of the variance parameter appears to be a challenge for existing methods. Table 4 replicates results from [38] with the parametrization of the model with the scale parameter μ, and Table 5 presents results for the parametrization with the scale parameter β and different data-generating processes. Both Monte Carlo studies are conducted with 1000 iterations. Our findings are comparable to those of [38]: the mean of the volatility process and the persistence parameter are estimated quite accurately, while the variance of the latent volatility process estimated with INLAis biased—usually, it is overestimated. Second, we provide the posterior moments for INLA similarly to Table 3 for PMH and RMHMC. These results are presented in Table 6. These results also suggest that the variance of the latent volatility process tends to be overestimated with INLA to a larger degree than with RMHMC, which also overestimates this parameter, as can be seen from the Table 3. Moreover, highest posterior density intervals for the parameter ϕ obtained with INLA are larger than those obtained with PMH and RMHMC.

### 4.4. Fixed-Form Variational Bayes

**Figure 6:**
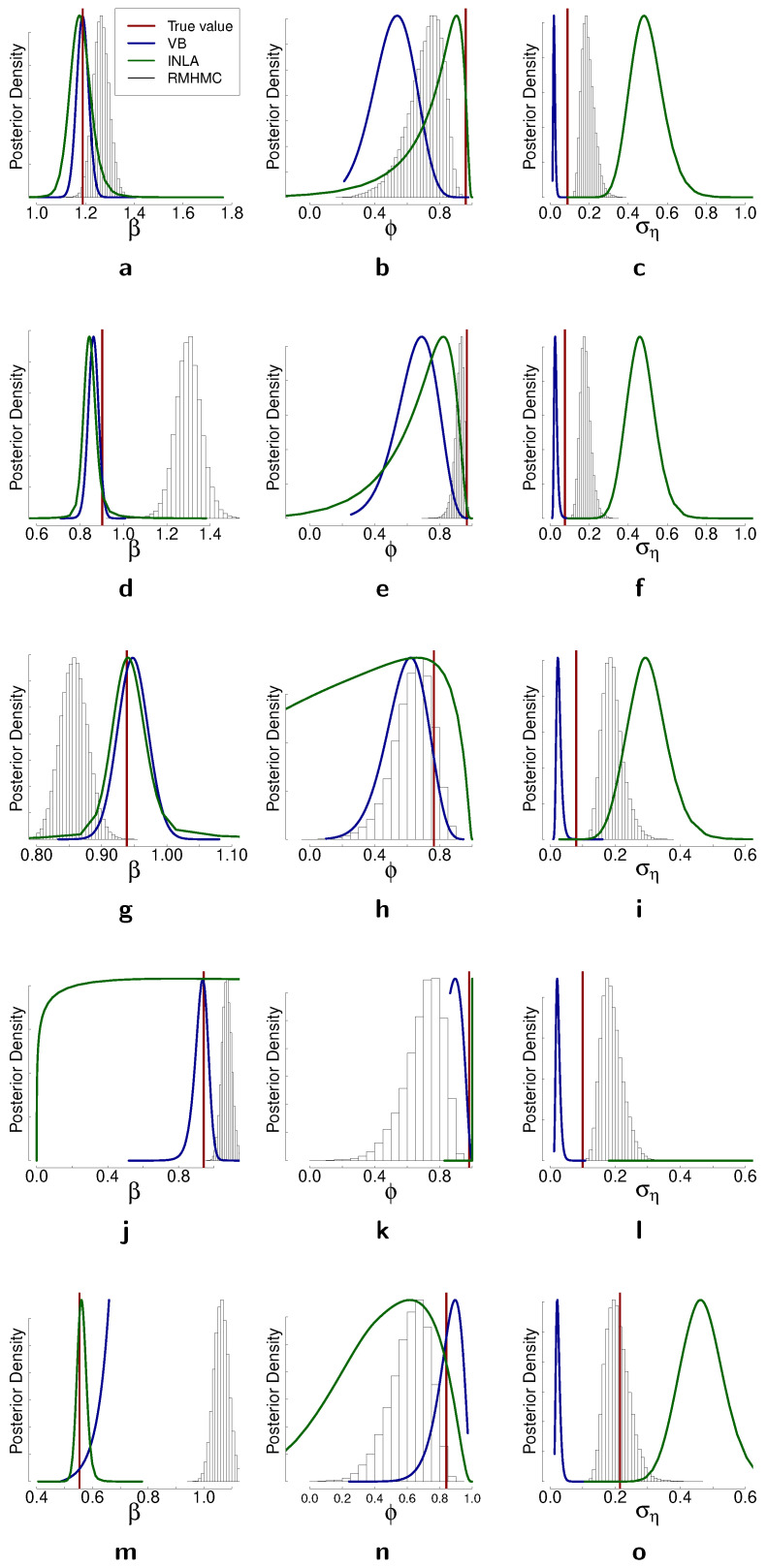
Illustration of the Fixed-form Variational Bayes in comparison to RMHMC and INLA. Subfigures illustrate the posterior distributions estimated with different methods for the different data-generating processes. (**a**–**c**) correspond to Experiment 1 from Table 2 and Table 6, (**d**–**f**) correspond to Experiment 2, (**g**–**i**) correspond to Experiment 3, (**j**–**l**) correspond to experiment 4, and (**m**–**o**) correspond to Experiment 5. Red vertical lines indicate true parameter values.

In this section, we discuss results for the simulation study with fixed-form variational Bayes. We consider the same time series as in the case of comparison between PMH and RMHMC. In Table 7, we present estimated variational parameters and in Figure 6, comparison of the posterior with fixed-form variational Bayes (in blue), RMHMC (histograms from the posterior samples), and INLA (green). It is clear that in some cases, the variational Bayes method performs quite well; in particular, parameter β is very well estimated in most of the cases. Only in Figure 6j is the approximate posterior for β far from the truth. The variance parameter is underestimated in all cases with VB; this is less severe in the cases when the true variance is relatively small. However, when the true variance is relatively large, the discrepancy between VB estimate and the true value increases, as can be seen from Figure 6o. We observe the opposite picture with INLA: it tends to overestimate the variance of the latent volatility process. Overestimation of the variance of the latent volatility process for stochastic volatility models with INLA has been previously reported in [38]. Additionally, it is reported in [38] that this effect decreases with larger values of $\sigma_{\eta }$. The source of this has to be investigated further. RMHMC overestimates the variance to a lesser degree than INLA, and as can be seen from Figure 6o, this is also connected to the value of the ground truth for $\sigma_{\eta }$: with larger true value of $\sigma_{\eta }$, RMHMC provides more accurate results.

### 4.5. Comparison of the Methods on the Real Data

**Figure 7:**
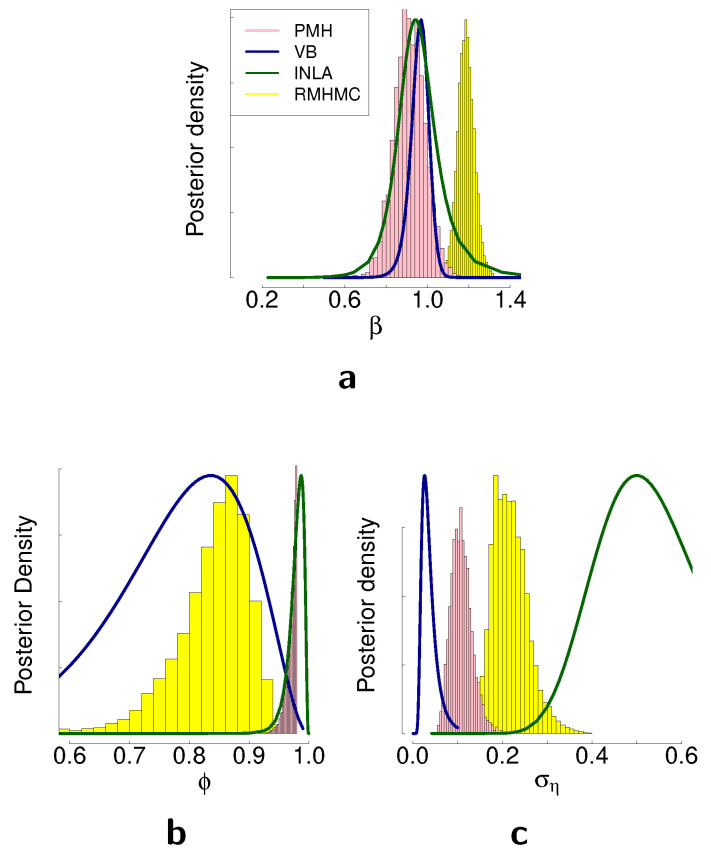
Comparison of PMH (pink), VB (blue), INLA (green), and RMHMC (yellow) on the weekly log-returns of the Australian dollar against the US dollar. Subfigures illustrate the posterior distributions for different parameters of the model obtained with different methods. (**a**) Corresponds to the posterior distribution for the parameter β. (**b**) Corresponds to the posterior distribution of the parameter ϕ. (**c**) Corresponds to the posterior distribution of the parameter $\sigma_{\eta }$.

**Figure 8:**
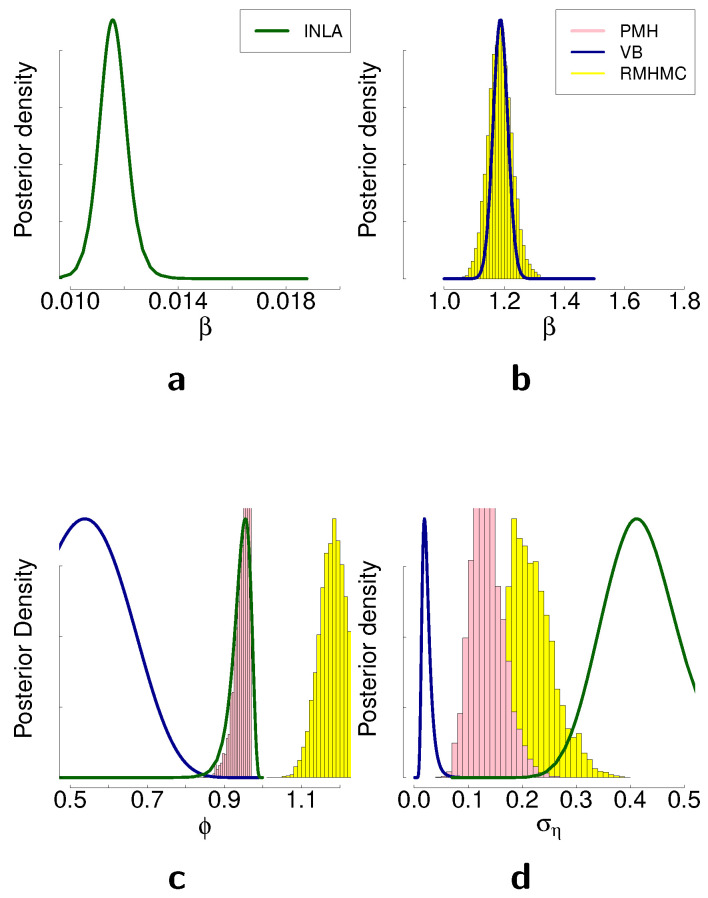
Comparison of PMH (pink), VB (blue), INLA (green), and RMHMC (yellow) on the daily log-returns of DAX index. Subfigures illustrate the posterior distributions for different parameters of the model obtained with different methods. (**a**,**b**) correspond to the posterior distribution of the parameter β. (**c**) corresponds to the posterior distribution of the parameter ϕ. (**d**) corresponds to the posterior distribution of the parameter $\sigma_{\eta }$.

In this section, we present posterior distributions of the parameters estimated with different Bayesian inference methods on two real-world time series. First, we consider the mean corrected log-returns of the Australian dollar against the US dollar. The data range from January 1994 to December 2003 with a total of 519 weekly observations. Resulting posterior distributions obtained with different inference methods are presented in Figure 7. Second, we consider daily log-returns for the DAX index from 3 January 2000 until 17 May 2001, which in total constitute 1000 observations. We provide descriptive statistics for both time series in Table A2. Resulting posterior distributions for this time series are presented in Figure 8. The main discrepancies between the methods are largest in the estimation of the parameter $\sigma_{\eta }$ for both time series, and the results are consistent with the simulation studies in terms of the difference of these discrepancies. As we can see from Figure 7c and  Figure 8d, the posterior distribution of $\sigma_{\eta }$ obtained with variational Bayes is concentrated in smaller values in comparison to the other methods. INLA suggests the higher values for $\sigma_{\eta }$ in comparison to the other methods. The posterior samples obtained with RMHMC are concentrated in values higher than the ones obtained with PMH. Both sampling methods appear to give results larger than VB but smaller than INLA for the parameter $\sigma_{\eta }$.

In Table 8, we present results for efficient sample size (ESS) for both empirical applications and both samplers. Similarly to what is found in simulation studies, ESS is higher in the case of the PMH algorithm. However, if the computational time were taken into account, this advantage would have disappeared, similarly to the results in Table 2.

## 5. Discussion

This paper reviewed multiple methods for the estimation of nonlinear state-space modes and stochastic volatility modes in particular that appear in Bayesian statistics and machine learning. We in particular focused on representative inference methods from different classes: methods that can recover the posterior distribution ‘exactly’ and the ones that build an approximation. We discussed which methods have the potential to be applied in a multivariate or high-dimensional situation and why they have this potential. Finally, we discovered that while stochastic volatility models are common for use in simulation studies for demonstrating the performance of the methods, usually not enough possible data-generating processes are considered to make a fair comparison. In particular, the performance of the methods is heavily connected to the variance of the latent volatility process.

State-space models can be powerful tools for modeling latent variables in different scientific fields. However, already for univariate time series, they are challenging to estimate. This paper’s main aim was to review and understand the existing classes of methods of estimation (targeting exact posterior or approximating it) and define the direction one can undertake for the estimation of *multivariate* nonlinear state-space models. The challenge arises from both statistical and computational perspectives. By this, we mean it is hard to develop methods that both provide sufficiently good results from the estimation point of view and are computationally feasible. We have reviewed a number of methods that allow a trade-off between these two aspects. In particular, we have considered particle Markov Chain Monte Carlo and reviewed multiple particle filtering approaches for this method, Riemann Manifold Langevin Hamiltonian Monte Carlo, Integrated Nested Laplace Approximation, and Variational Bayes. All these methods are equipped with the ability to estimate models with intractable likelihoods.

### 5.1. Sequential Monte Carlo

We compared the auxiliary particle filter with the bootstrap particle filter in terms of the variance of the estimated likelihood. We found that the auxiliary particle filter outperformed the bootstrap particle filter for most of the data-generating processes. As discussed in [27], auxiliary particle filter does not always have a smaller variance of the estimated likelihood. Additionally, we looked into how the variance of the estimated likelihood changes in the parameter space. We found that, in particular, the variance of the latent process affects the variance of the estimated likelihood. This implies that one has to find the balance for the number of particles used in Sequential Monte Carlo and a clever way of finding initial parameter values for the sampling from the posterior, especially when considering multivariate models. The advantage of the auxiliary particle filter from the methodological point of view is that it takes into account current observation $y_{t}$ when constructing the proposal for the particles $q(h_{t}|h_{t-1},y_{t})$. The method that we did not include in our simulation study, but that possibly can solve the problem with the variance of the estimated likelihood, is the iterated auxiliary particle filter (iAPF): for the proposal of the particles, it uses not only current observation $y_{t}$, but all observations $q(h_{t}|h_{t-1},y_{1:T})$. A backward sequential procedure with an optimization step is used in this proposal mechanism for the particles, which makes the algorithm computationally intensive. The multivariate application of the stochastic volatility model in [26] considers only diagonal case of the matrix Φ, and the proposed procedure for the particle proposals does not incorporate such dependence. While this method does introduce an additional computational burden on already computationally intensive method (particle Metropolis–Hastings), it is promising for getting state-of-the-art results for the task of parameter estimation.

### 5.2. Particle Metropolis-Hastings

Metropolis-Hastings is a general MCMC method that is easy to implement and works well for the univariate model. The estimation results are satisfying when it is properly calibrated, and good mixing of the chains is achieved. It works well in low-dimensional problems but is unlikely to be successful in the case of multivariate stochastic volatility models. Considering the non-diagonal matrix Φ in a five-dimensional case, we would have 45 parameters to be estimated. The random walk proposal would be very inefficient even with a reasonable sparseness assumption on Φ. Nevertheless, in the low-dimensional model, we get the best estimation results with particle Metropolis–Hastings, where the particle filtering scheme is chosen to be an auxiliary particle filter. From the methods considered in this paper, particle Markov Chain Monte Carlo methods are easiest to adapt to different specifications of the model and are easiest to implement.

### 5.3. Riemann Manifold Hamiltonian Monte Carlo Methods

Hamiltonian Monte Carlo is a very attractive method for high-dimensional problems as it allows us to explore the parameter space efficiently. In particular, the gain in efficiency comes from avoiding random walk behavior in the proposals. The disadvantage comes from the need of careful calibration since there is no principled way of choosing matrix M. RMHMC avoids this problem by exploiting underlying geometry in the proposal mechanism. In our study, we notice that RMHMC results in good mixing of the Markov chains, and the method is generally easy to calibrate, but the estimation of the parameters is not very good. In particular, it appears that the variance of the latent volatility process is challenging for the method. It is not surprising that the PMH algorithm performs better in terms of parameter estimation since we use an auxiliary particle filter for the volatility process estimation and thus take current observation $y_{t}$ for the particle proposals. RMHMC does not benefit from similar information when estimating model parameters. Therefore, improved estimation of the volatility process can be one of the directions for improving the performance of RMHMC for the parameter estimation of stochastic volatility models.

### 5.4. Variational Bayes

As one can see from the illustrative example, in some cases, variational Bayes performs quite well; however, there are also situations when it is far off from the underlying truth. The challenge with stochastic volatility models remains the same: it is difficult to estimate the latent states. In the approach of [6], this is done via Kalman filtering. Therefore, the drawback of linearization of the model will remain and will show in the final results. In this respect, the possible combination of VB and SMC can be of interest. Some advances in this direction have already been made [42].

### 5.5. Integrated Nested Laplace Approximation

Integrated Nested Laplace Approximation is another approach that works well considering how fast the method is, but it clearly overestimates the variance of the latent volatility process. Additionally, the sparse matrix computation that is used in univariate models is not applicable to the multivariate case. In the multivariate case, the precision matrix in Equation (67) is not sparse, and thus, the method does not benefit from fast sparse matrix computation. An approach that we have not considered in this paper is the Expectation Propagation algorithm. In particular, the authors of [43] propose a way to improve approximate marginals $p(x_{t}|\theta ,y)$ in latent Gaussian fields by using EP. The motivation of the approach builds on the fact that EP can give better approximations than the Laplace approximation in this case. The improvements, however, would come at computational costs. In the univariate case, the extra computational costs do not play a significant role as the algorithm can be parallelized. However, it is hard to say how big the difference would be in the multivariate model, both in terms of improvement in the estimation and loss in computational speed.

## 6. Conclusions

We reviewed multiple Bayesian inference methods, which both target the exact posterior distribution and approximate it. By comparing methods on various data-generating processes, we notice that variational Bayes tends to underestimate the latent volatility process variance, while INLA and RMHMC, in the cases considered, overestimated this parameter. We also get similar disposition of the results on two real-world data sets. We achieved the best performance with PMH in terms of recovering ground truth and uncertainty quantification. In PMH, the particle filtering step was performed with an auxiliary particle filter. This indicates that filtering with look-ahead approaches, which include current (or future) observations into proposal machinery can improve the performance of the inference method. It is important to note that different data-generating processes for simulation studies would indicate different performance results. Thus, we stress that when using stochastic volatility models, more than one data-generating process should be considered for methods comparison. This practice would allow indicating in which situation a method can fail or perform differently. Our results indicate that fixed-form variational Bayes tends to underestimate the variance of the latent process, while RMHMC and INLA overestimated this parameter. To estimate the stochastic volatility model in the multivariate case, the combination of different strategies appears to be necessary. In a high-dimensional case, the random-walk proposal would become extremely inefficient. At the same time, approximate methods lose their outstanding computational advantage (for example, INLA), and the implementation of these methods in the multivariate case is not straightforward.

## Figures and Tables

**Table 1:** Variance, bias, and number of effective particles $N_{eff}$ for the estimated likelihood with bootstrap particle filter (BPF) and auxiliary particle filter (APF) averaged over 50 time series. $N_{eff}$ is computed as in Equation (22). Variance and bias are computed as in Equations (93) and  (94).

	Variance		Bias		$N_{\mathrm{eff}}$	
	**BPF**	**APF**	**BPF**	**APF**	**BPF**	**APF**
*N* = 100	778.46	3.36	19.95	−0.68	64.07	23.52
*N* = 1000	805.79	0.20	20.32	−0.07	640.23	224.65
*N* = 10,000	808.40	0.02	20.36	−0.01	6402.11	2233.59

**Table 2:** Variance of the estimated likelihood for 10 different data-generating processes (TS). We consider different settings for the number of particles *N*.

Bootstrap Particle Filter				Auxiliary Particle Filter			True Parameters		
TS	N = 100	N = 1000	N = 10,000	N = 100	N = 1000	N = 10,000	β	ϕ	$\sigma_{\eta }$
1	511.26	556.01	559.85	2.436	0.251	0.017	0.36	0.96	0.13
2	128.50	138.05	139.19	0.688	0.040	0.005	1.18	0.95	0.08
3	18,143.0	18,883.0	18,945.0	259.3	9.669	0.993	1.93	0.89	0.43
4	79.085	84.045	83.510	0.517	0.045	0.005	0.9	0.96	0.07
5	51.753	52.112	52.078	0.066	0.005	0.001	0.93	0.76	0.08
6	4.2531	4.2704	4.2961	0.026	0.003	0.000	1.43	0.79	0.04
7	2684.2	2780.2	2790.5	5.076	0.339	0.032	1.58	0.91	0.27
8	137.22	140.65	140.48	0.210	0.018	0.002	0.09	0.85	0.11
9	8.5151	13.541	13.419	0.808	0.067	0.008	0.94	0.98	0.10
10	3601.2	3665.6	3670.4	25.32	2.614	0.291	0.55	0.84	0.21

**Table 3:** Posterior moments for the samples obtained with particle Metropolis–Hastings (PMH) and Riemann Manifold Hamiltonian Monte Carlo (RMHMC) for the parameters β, ϕ and $\sigma_{\eta }$ of the stochastic volatility model. Experiments 1, 2, 3, 4, and 5 correspond to TS 2, 4, 5, 9, and 10 from Table 2.

**Experiment 1: Posterior Moments Obtained with PMH**							
	Mean	Mode	95% $HPD_{l}$	95% $HPD_{u}$	True	ESS	ESS/s
β	1.1985	1.2140	1.0771	1.3034	1.189	2783.2	0.054
ϕ	0.9713	0.9732	0.9392	0.9987	0.959	1516.8	0.029
$\sigma_{\eta }$	0.0537	0.0514	0.0260	0.0828	0.089	2280.7	0.044
**Experiment 1: Posterior Moments Obtained with RMHMC**							
	Mean	Mode	95% $HPD_{l}$	95% $HPD_{u}$	True	ESS	ESS/s
β	1.18628	1.16608	1.10609	1.26526	1.189	3580.7	50.57
ϕ	0.83824	0.78390	0.71272	0.93643	0.959	188.6244	2.66
$\sigma_{\eta }$	0.21889	0.17203	0.14846	0.30072	0.0828	93.5265	1.32
**Experiment 2: Posterior Moments Obtained with PMH**							
	Mean	Mode	95% $HPD_{l}$	95% $HPD_{u}$	True	ESS	ESS/s
β	0.8629	0.8780	0.7870	0.9385	0.903	2909.6	0.045
ϕ	0.9644	0.9843	0.9077	0.9987	0.967	1157.2	0.017
$\sigma_{\eta }$	0.0534	0.0504	0.0233	0.0912	0.076	1694.3	0.026
**Experiment 2: Posterior Moments Obtained with RMHMC**							
	Mean	Mode	95% $HPD_{l}$	95% $HPD_{u}$	True	ESS	ESS/s
β	0.85071	0.82888	0.79927	0.90418	0.903	4108.8	76.26
ϕ	0.79293	0.73992	0.62454	0.92638	0.967	186.8240	3.46
$\sigma_{\eta }$	0.22291	0.23163	0.14751	0.30825	0.076	72.6236	1.34
**Experiment 3: Posterior Moments Obtained with PMH**							
	Mean	Mode	95% $HPD_{l}$	95% $HPD_{u}$	True	ESS	ESS/s
β	0.9579	0.9623	0.9090	1.0043	0.938	3330.7	0.071
ϕ	0.9079	0.9583	0.7974	0.9885	0.764	2178.9	0.046
$\sigma_{\eta }$	0.0429	0.0317	0.0184	0.0725	0.08	2690.1	0.057
**Experiment 3: Posterior Moments Obtained with RMHMC**							
	Mean	Mode	95% $HPD_{l}$	95% $HPD_{u}$	True	ESS	ESS/s
β	0.94395	0.90574	0.89572	0.99246	0.938	8112.2	164.83
ϕ	0.67333	0.43245	0.42649	0.88097	0.764	116.9159	2.37
$\sigma_{\eta }$	0.19759	0.18142	0.13385	0.27098	0.08	98.9095	2.0
**Experiment 4: Posterior Moments Obtained with PMH**							
	Mean	Mode	95% $HPD_{l}$	95% $HPD_{u}$	True	ESS	ESS/s
β	0.8865	0.8932	0.7297	1.0485	0.942	3046.7	0.057
ϕ	0.9826	0.9892	0.9672	0.9961	0.981	1781.9	0.033
$\sigma_{\eta }$	0.1100	0.1011	0.0702	0.1503	0.1	1609.9	0.031
**Experiment 4: Posterior Moments Obtained with RMHMC**							
	Mean	Mode	95% $HPD_{l}$	95% $HPD_{u}$	True	ESS	ESS/s
β	0.90560	0.80476	0.74840	1.07499	0.942	445.7	9.22
ϕ	0.96391	0.96149	0.93865	0.98672	0.981	336.5	6.96
$\sigma_{\eta }$	0.17588	0.15766	0.13452	0.22005	0.1	130.0	2.69
**Experiment 5: Posterior Moments Obtained with PMH**							
	Mean	Mode	95% $HPD_{l}$	95% $HPD_{u}$	True	ESS	ESS/s
β	0.5836	0.5893	0.5359	0.6293	0.553	2733.8	0.0552
ϕ	0.9497	0.9970	0.8890	0.9970	0.840	2733.8	0.0552
$\sigma_{\eta }$	0.0756	0.0525	0.0294	0.1331	0.215	1589.1	0.032
**Experiment 5: Posterior Moments Obtained with RMHMC**							
	Mean	Mode	95% $HPD_{l}$	95% $HPD_{u}$	True	ESS	ESS/s
β	0.56805	0.57215	0.53372	0.60258	0.553	3276.6	75.41
ϕ	0.77895	0.79364	0.60134	0.92411	0.840	130.7	3.0
$\sigma_{\eta }$	0.22905	0.21974	0.14799	0.31755	0.215	64.9	1.49

**Table 4:** Bias and square root of the mean squared error for integrated nested Laplace approximation (INLA) parametrized with scale parameter μ.

$\mu_{\mathrm{true}}$	$\phi_{\mathrm{true}}$	$\sigma_{\eta \mathrm{true}}$	bias (μ)	smse (μ)	bias (ϕ)	smse (ϕ)	bias ($\sigma_{\eta }$)	smse ($\sigma_{\eta }$)
0.1366	0.9	0.0186	−0.0672	0.01804	−0.6138	0.5403	0.29183	0.0912
−0.2143	0.9	0.0366	−0.0565	0.0092	−0.5803	0.4953	0.2739	0.0815
−0.0658	0.9	0.0636	−0.0664	0.0151	−0.5817	0.5176	0.2494	0.0704
−0.0289	0.95	0.0186	−0.0675	0.0151	−0.6354	0.5550	0.2883	0.0900
0.0203	0.95	0.0366	−0.0705	0.0207	−0.5796	0.4843	0.2677	0.0795
−0.0630	0.95	0.0636	−0.0931	0.0333	−0.4142	0.3250	0.2129	0.0592
−0.0174	0.98	0.0186	−0.0763	0.0222	−0.6173	0.5458	0.2788	0.0874
0.1343	0.98	0.0366	−0.1534	0.0797	−0.4329	0.3650	0.2228	0.0654
0.0584	0.98	0.0636	−0.2596	0.1379	−0.2095	0.1710	0.1280	0.034

**Table 5:** Bias and square root of the mean squared error for INLA parametrized with scale parameter β.

$\beta_{\mathrm{true}}$	$\phi_{\mathrm{true}}$	$\sigma_{\eta \mathrm{true}}$	bias (μ)	smse (μ)	bias (ϕ)	smse (ϕ)	bias ($\sigma_{\eta }$)	smse ($\sigma_{\eta }$)
0.367	0.965	0.134	−0.2295	0.0526	−0.0083	0.0001	0.4725	0.2233
1.188	0.959	0.088	0.2000	0.0400	−0.1909	0.0364	0.3466	0.1201
1.937	0.897	0.433	1.6162	2.6122	−0.0021	0.0000	0.5926	0.3512
0.902	0.966	0.075	−0.1888	0.0356	−0.3049	0.0930	0.3153	0.0994
0.938	0.764	0.080	−0.0515	0.0026	−0.4921	0.2421	0.1468	0.0215
1.435	0.793	0.048	0.6014	0.3616	−0.3872	0.1499	0.2155	0.0464
1.588	0.919	0.275	0.2539	0.0644	0.0202	0.0004	0.4786	0.2291
0.092	0.857	0.109	−0.0841	0.0070	−0.7490	0.5610	0.1341	0.0179
0.942	0.980	0.100	−0.3246	0.1054	0.0192	0.0003	95.8384	9185. 0
0.553	0.840	0.214	−0.2384	0.0568	−0.3904	0.1524	0.1918	0.0368

**Table 6:** Posterior results for estimation of the stochastic volatility (SV) model with INLA. Experiments 1, 2, 3, 4, and 5 correspond to TS 2, 4, 5, 9, and 10 from Table 2.

**Experiment 1**					
	mean	mode	95% $HPD_{l}$	95% $HPD_{u}$	true
β	1.1786	1.1753	1.0937	1.2797	1.189
ϕ	0.7717	0.9986	0.2029	0.9945	0.959
$\sigma^{\eta }$	0.4368	0.4918	0.3048	0.6665	0.089
**Experiment 2**					
	mean	mode	95% $HPD_{l}$	95% $HPD_{u}$	true
β	0.8457	0.8426	0.7807	0.9367	0.903
ϕ	0.7480	0.9993	−0.0844	0.9996	0.967
$\sigma_{\eta }$	0.4155	0.9111	0.2631	0.7726	0.076
**Experiment 3**					
	mean	mode	95% $HPD_{l}$	95% $HPD_{u}$	true
β	0.9446	0.9403	0.8672	1.0798	0.938
ϕ	0.3827	0.9999	−0.7491	0.9999	0.764
$\sigma_{\eta }$	0.2328	4.5772	0.1327	0.5202	0.080
**Experiment 4**					
	mean	mode	95% $HPD_{l}$	95% $HPD_{u}$	true
β	0.0892	0.0893	0.0847	0.0939	0.942
ϕ	0.1266	1.0000	−0.9470	0.9994	0.981
$\sigma_{\eta }$	0.2405	0.3196	0.1407	0.4447	0.100
**Experiment 5**					
	mean	mode	95% $HPD_{l}$	95% $HPD_{u}$	true
β	0.5614	0.5604	0.5286	0.5993	0.553
ϕ	0.4567	0.9980	−0.3055	0.9726	0.840
$\sigma_{\eta }$	0.4067	0.4538	0.2888	0.5680	0.215

**Table 7:** Parameters of the posterior distribution obtained with fixed-form Variational Bayes. Exp. 1–5 correspond to the Experiments 1–5 in Table 3 and Table 6.

Exp.	$\xi_{1}$	$\xi_{2}$	$\xi_{3}$	$\xi_{4}$	$\xi_{5}$
1	31.1347	9.3573	19.1852	0.4658	−0.2051
2	28.2720	5.2039	12.6658	0.4510	−0.1812
3	30.3353	7.1038	13.9647	0.4563	−0.2039
4	38.8151	2.1501	17.9035	0.7568	−0.4296
5	38.8151	2.1501	17.9035	0.7568	−0.4296

**Table 8:** Efficient sample size (ESS) for PMH and RMHMC in real-world time series applications: weekly log-returns for the exchange rate of Australian/US dollars and daily log-returns of DAX index.

**Australian/US Dollars Exchange Rate**		
	ESS PMH	ESS RMHMC
β	3373.9	906.2
ϕ	1546.4	481
$\sigma_{\eta }$	2439	208.3
**DAX Index**		
	ESS PMH	ESS RMHMC
β	3868.4	3962.3
ϕ	915.3	134.6
$\sigma_{\eta }$	2439	79.6

## Data Availability

Not applicable.

## Abbreviations

   The following abbreviations are used in this manuscript:

SV	Stochastic volatility
SMC	Sequential Monte Carlo
iAPF	Iterated Auxiliary Particle Filter
PMCMC	Particle Markov Chain Monte Carlo
PMH	Particle Metropolis-Hastings
HMC	Hamiltonian Monte Carlo
RMHMC	Riemann Manifold Hamiltonian Monte Carlo
VB	Variational Bayes
INLA	Integrated Nested Laplace Approximation

## Appendix A

### Appendix A.2.

Although we do not give details for the NUTS sampler [44] in the main text, we present here experiments for this sampler using the same experiments as in the main text. The results in Table A1 are obtained with the sampler implemented in RStan [45]. The method provides large confidence intervals for the parameters ϕ and $\sigma_{\eta }$. Similarly to RMHMC, the variance of the latent volatility process tends be overestimated based on the mean and the mode of the posterior distribution. The confidence intervals obtained with NUTS appear to be quite large, especially for the parameters ϕ and $\sigma_{\eta }$. The multivariate version of the stochastic volatility model can provide additional challenges since different parameters might require different step sizes and the sampler can get stuck in the regions of space where a small step size is needed to achieve target acceptance rate. In the univariate case, NUTS appears to be more efficient than both PMH and RMHMC as can be seen from the last two columns of Table A1. The applicability of this particular implementation can be limited due to the large 95% highest posterior intervals as uncertainty about parameters ϕ and $\sigma_{\eta }$ is very large in most cases.

**Table A1:** Posterior results for estimation of the SV model with INLA. Experiments 1, 2, 3, 4, and 5 correspond to TS 2, 4, 5, 9, and 10 from Table 2.

**Experiment 1**							
	mean	mode	95% $HPD_{l}$	95% $HPD_{u}$	true	ESS	ESS/s
β	1.1785	1.1779	1.1053	1.2567	1.189	18794	87.31
ϕ	0.6266	0.8366	0.0886	0.9773	0.959	18704	86.89
$\sigma_{\eta }$	0.3117	0.2985	0.0961	0.5397	0.089	18534	86.10
**Experiment 2**							
	mean	mode	95% $HPD_{l}$	95% $HPD_{u}$	true	ESS	ESS/s
β	0.8498	0.8509	0.7978	0.9004	0.903	18694	42.08
ϕ	0.4033	0.7162	−0.3309	0.9912	0.967	17888	40.26
$\sigma_{\eta }$	0.2944	0.3064	0.0547	0.5127	0.076	18789	42.29
**Experiment 3**							
	mean	mode	95% $HPD_{l}$	95% $HPD_{u}$	true	ESS	ESS/s
β	0.9510	0.9510	0.9074	0.9960	0.938	19001	42.31
ϕ	0.0188	−0.2263	−0.8129	0.8727	0.764	18168	40.46
$\sigma_{\eta }$	0.1259	0.0276	0.0000	0.2906	0.080	19001	42.31
**Experiment 4**							
	mean	mode	95% $HPD_{l}$	95% $HPD_{u}$	true	ESS	ESS/s
β	0.9096	0.9042	0.7178	1.1550	0.942	18587	39.88
ϕ	0.9753	0.9784	0.9525	0.9965	0.981	18529	39.75
$\sigma_{\eta }$	0.1398	0.1317	0.0876	0.1923	0.100	18397	39.47
**Experiment 5**							
	mean	mode	95% $HPD_{l}$	95% $HPD_{u}$	true	ESS	ESS/s
β	0.5631	0.5632	0.5329	0.5958	0.553	18819	42.40
ϕ	0.2804	0.3784	−0.3789	0.8847	0.840	18703	42.14
$\sigma_{\eta }$	0.3648	0.3910	0.1657	0.5445	0.215	18248	41.11

### Appendix A.3.

Algorithm A1 is a generic particle filter. We use auxiliary version of it proposed in [23].

**Algorithm A1** Approximation of marginal likelihood with ASIR algorithm.
1: Draw *N* samples $h_{0}^{\left(i\right)}$ from the prior (A1) $$h_{0}^{\left(i\right)}∼\pi \left(h_{0}|\theta \right),\;\;\;i=1,\cdots ,N$$ and set $w_{0}^{\left(i\right)}=1/N$, for all i=1,⋯,N. 2: For each t=1,⋯,T do the following 3: Draw samples $h_{t}^{\left(i\right)}$ from the importance distribution (A2) $$h_{t}^{\left(i\right)}∼q\left(h_{t}|h_{t-1}^{\left(i\right)},y_{1:t},\theta \right),\;\;\;i=1,\cdots ,N.$$ 4: Compute the following weights (A3) $$w_{t}^{\left(i\right)}=\frac{g\left(y_{t}|h_{t}^{\left(i\right)},\theta \right)f\left(h_{t}^{\left(i\right)}|h_{t-1}^{\left(i\right)},\theta \right)}{q(h_{t}^{\left(i\right)}|h_{t-1}^{\left(i\right)},y_{1:t},\theta )}$$ and compute the estimate of $p(y_{t}|y_{1:t-1},\theta )$ as (A4) $$\hat{p}\left(y_{t}|y_{1:t-1},\theta \right)=\sum_{i}W_{t-1}^{\left(i\right)}w_{t}^{\left(i\right)}.$$ 5: Compute normalized weights as (A5) $$W_{t}^{\left(i\right)}\propto W_{t-1}^{\left(i\right)}w_{t}^{\left(i\right)}.$$ 6: If the effective number of particles is too low, perform resampling.

### Appendix A.4.

**Table A2:** Descriptive statistics for time series from the empirical example in Section 3.5: daily log-returns for DAX index and weekly log-returns for Australian/US dollar exchange rate.

	DAX	Australia/US
Mean	−0.001	−0.04
Std.dev.	0.013	1.00
Skewness	0.202	0.06
Kurtosis	3.253	3.37
